# The application of Tong-fu therapeutic method on ulcerative colitis: A systematic review and meta-analysis for efficacy and safety of rhubarb-based therapy

**DOI:** 10.3389/fphar.2022.1036593

**Published:** 2022-10-21

**Authors:** Yuzheng Li, Zhen Ye, Haiqing He, Yu Hu, Mingquan Wu, Linzhen Li, Liulin Chen, Huanzhu Qian, Qingyu Shi, Chen Zhang, Han Yu, Qian Zhao, Xinglong Liu, Kaihua Qin, Qiaobo Ye

**Affiliations:** ^1^ School of Basic Medical Sciences, Chengdu University of Traditional Chinese Medicine, Chengdu, China; ^2^ Department of Pharmacy, Sichuan Orthopedic Hospital, Chengdu, China; ^3^ School of Clinical Medicine, Chengdu University of Traditional Chinese Medicine, Chengdu, China; ^4^ State Key Laboratory of Southwestern Chinese Medicine Resources, Chengdu University of Traditional Chinese Medicine, Chengdu, China; ^5^ Health Preservation and Rehabilitation College, Chengdu University of Traditional Chinese Medicine, Chengdu, China

**Keywords:** rhubarb, *dà huáng*, Tong-fu therapeutic method, traditional Chinese medicine, metaanalysis, ulcerative colitis

## Abstract

**Background**: Tong-fu therapeutic method (TFTM) is a traditional Chinese medicine treatment method for ulcerative colitis, which is a novel treatment strategies and have purgative effect. As the most representative medicinal of TFTM, Rhubarb has been reported to have a therapeutic impact on ulcerative colitis by regulating intestinal flora, anti-inflammation, and improving intestinal microcirculation. Although rhubarb has been widely used in Chinese medicine for the treatment of ulcerative colitis, the appropriate protocol is still demanded to its rational use in clinic, which promoted to evaluate the efficacy and safety for rhubarb-based therapy on ulcerative colitis.

**Method**: Clinical trials were searched through PubMed, Cochrane Library, Web of Science, Excerpta Medica Database, Chinese National Knowledge Infrastructure, WAN FANG Database, Chinese Scientific Journal Database, and Chinese Biomedical Literature Database. The subgroup analyses were performed with three groups: medication, course of treatment, and route of administration. The statistical analyses were performed on Review Manager software (version 5.4.1).

**Results**: A total of 2, 475 patients in 30 original studies were analyzed in this article. It was found that rhubarb-based therapy could increase clinical efficacy and reduce the recurrence rate. Subgroup analyses showed that rhubarb-based therapy was more effective than 5-aminosalicylic acid or sulfasalazine alone. In addition, the hypercoagulable state of ulcerative colitis could be ameliorated by decreasing platelet (PLT) and fibrinogen (FIB), and increasing prothrombin time (PT) significantly. Moreover, C-reaction protein (CRP), tumor necrosis factor-α (TNF-α), interleukin (IL)-6, IL-8, and IL-1β expression were significantly reduced, while IL-10 production was increased, which mediated the alleviation of intestinal inflammation stress.

**Conclusion**: Rhubarb-based therapy could effectively improve ulcerative colitis. Of note, the rhubarb-based medicinal formulas combined with 5-ASA or SASP are more effective than the 5-ASA or SASP alone. In addition, although rhubarb has side effect, the results of our analysis showed that rhubarb-based therapy did not exhibit significant side effects. This means it has a high safety profile in clinical use. Moreover, the use of rhubarb-based therapy is recommend to use within 1–13 weeks or 3 months *via* administered orally or by enema, which is contributes to ensure the curative effect and avoid its toxic and side effects. As an important case of TFTM, rhubarb-based therapy provides evidence for the practical application of TFTM.

## Introduction

Ulcerative colitis (UC) is a subtype of inflammatory bowel disease (IBD). The prevalence of IBD in the western world has reached as high as 0.5% of the total population (Kaplan, 2015). UC has evolved into a global burden given its high incidence in developed countries and the substantial increase in incidence in developing countries (Sood et al., 2003; Molodecky et al., 2012; Kobayashi et al., 2020). Even though the pathogenesis of UC has not been revealed ultimately, immunological abnormalities are considered to play an essential role, and drugs regulating the innate immune system have become the mainstream treatment. 5-aminosalicylic acid (5-ASA), corticosteroids, and thiopurines can effectively alleviate UC as first-line therapeutic agents (Kobayashi et al., 2020). However, several limitations of the treatments have attracted increasing attention. 5-ASA is beneficial to mild to moderate UC but undesirable for severe UC. Meanwhile, corticosteroids, as a supplement to 5-ASA, are considered to lack long-term efficacy and safety. The application of thiopurines is challenged with increasing evidence of potential serious adverse reactions such as bone marrow and liver toxicity, pancreatitis, increased risk of non-melanoma skin cancer and lymphoma (Ko et al., 2019; Kobayashi et al., 2020). Due to the lack of satisfactory treatment, UC patients relapse continually even after routine treatment (Kobayashi et al., 2020). In essence, more effective treatment options with less adverse reactions still expected.

Tong-fu therapeutic method (TFTM) as an important traditional Chinese medicine therapy, can produce purgative effect and restore gastrointestinal motility. According to traditional Chinese medicine (TCM) theory, TFTM can descend qi, which discharges toxic and harmful products, breaks the vicious circle caused by toxin retention, improves intestinal microcirculation, and regulates the internal environment of the host, to treat and prevent the deterioration of the disease. Study has demonstrated that TFTM, whenever administered orally, rectally, or by nasal feeding, can improve gastrointestinal dysfunction and reduce case-fatality rate (Zhang et al., 2021). Rhubarb (*Rheum palmatum* L.) [*dà huáng*; Polygonaceae; *Rheum palmatum* root and rhizome], as one of the most representative medicinals of TFTM, was first recorded in *Shen Nong’s Classic of the Materia Medica* (*Shén Nóng Bĕn Căo Jīng*), which can play a powerful role in TFTM and improve digestive diseases (Teng et al., 2019). It was referred that rhubarb combined with conventional medication (somatostatin or trypsin inhibitor) in the treatment of acute pancreatitis was superior to conventional medication alone (Zhou et al., 2016; Hu et al., 2018). Also, rhubarb could modulate gut microbiota, which indirectly changed purine Metabolism in the intestine and subsequently alleviated DSS-induced chronic colitis (Wu et al., 2020). Additionally, rhubarb acted as an anti-platelet (PLT) aggregator that could significantly alleviate the hypercoagulable state associated with UC (Gao et al., 2020). In general, the rhubarb-based therapy is effective and authentic in improving UC, and its underlying mechanism appears to be multifaceted and meaningful. Presently, a large number preclinical and clinical evidence has clarified the positive effect of rhubarb and the rhubarb-based medicinal formulas on UC, however, the evidence is scattered. Because the influence of TFTM represented by rhubarb on UC has not been systematically supported by evidence-based medicine analysis, its scientific validity and potential clinical value have not been fully revealed. In this paper, a Meta-analysis of rhubarb-based therapy is conducted to assess the efficacy and safety. Meanwhile, the mechanisms of rhubarb improving hypercoagulable state of UC was systematically reviewed. Through the analysis and systematic review, it was given thata reliable application protocol of the rhubarb-based therapy could ensure the curative effect and its mechanism.

## Methods

This study was conducted following Preferred Reporting Items for Systematic Reviews and Meta-Analyses (PRISMA) statement (Moher et al., 2009). The requirements considered relevant in recent best practice guidelines for manuscripts of natural products have been taken into account (Heinrich et al., 2020).

### Search strategies

The search of the original studies was carried out until 7 October 2021 through Chinese National Knowledge Infrastructure (CNKI), the WANFANG Database, the Chinese Scientific Journal Database (VIP), the Chinese Biomedical Literature Database (CBM), Excerpta Medica Database (EMBASE), Web of Science, PubMed, and Cochrane Library. The medical subject headings were “ulcerative colitis” and “Rheum.”

The search formulation adopted a combination of free texts and medical subject headings (MeSH). According to the search habits of each database, the retrieval method that met the requirements was developed. The search formulas for each database are shown in Supplementary Data S1. At the same time, there is no restriction of search formulation on language or country.

### Study selection

#### Type of participants

In this study, all participants met the UC diagnostic criteria. As there is no gold standard for UC diagnosis, patients were subjected to comprehensive analysis, including symptom evaluation, endoscopic examination, and histopathological manifestations (Ungaro et al., 2017). Specifically, patients with a definite diagnosis of UC were included in the study and those with Crohn’s disease or unspecified IBD subtypes were excluded.

#### Type of interventions

Interventions in the treatment group were rhubarb-based therapeutic regimen, including rhubarb-based medicinal formulas alone or combined with UC routine treatment, including 5-ASA, sulfasalazine (SASP), olsalazine sodium, bifidobacterium triple viable capsules, glutamine, infliximab, prednisolone, hydrocortisone sodium succinate, and by oral or enema. Patients in the control group were treated with UC routine treatment. The trial was excluded if external TCM treatments such as acupuncture, massage, and moxibustion were present in the treatment group.

#### Type of outcome measures

Outcomes were divided into main outcome and secondary outcomes. The primary outcome was the clinical efficiency. The Mayo score, Geboes score, recurrence rate, PLT, adverse events, symptoms integral, and inflammatory cytokines and protein, including C-reaction protein (CRP), tumor necrosis factor-α (TNF-α), Interleukin (IL)-6, IL-8, IL-1β, and IL-10 were the secondary outcomes.

#### Type of studies

Randomized clinical trials (RCTs) were selected for Meta-analysis. Animal experiments, non-randomized controlled trials, and repeated data studies were excluded.

### Data extraction

Firstly, YL was responsible for formulating retrieval strategies and extracting retrieval records. Secondly, ZY and YH, and QZ independently screened the title and abstract of the articles, excluding the content that did not meet the standard. After combining the two results, YL, HH, and LC entered the full-text screening stage. When they encountered differences, the literatures were handed over to MW and CZ for judgment. In the end, from the included studies, information was extracted by two reviewers: HQ extracted the data, and YL was in charge of reviewing it.

### Assessment of the risk of bias

Based on the Cochrane Handbook, YL and HH independently assessed the bias risk of each study. The standard included the following seven items: *1*) sequence generation (selection bias); *2*) allocation concealment (selection bias); *3*) blinding of participants and personnel (performance bias); *4*) blinding of outcome assessments (detection bias); *5*) incomplete outcome data (attrition bias); *6*) selective reporting (reporting bias); *7*) other sources of bias. On the basis of methodological quality, the studies were classified as high risk, low risk, and unclear risk in each domain.

### Data analysis

Meta-analysis was performed by Review Manager (V.5.4.1 Copenhagen: The Nordic Cochrane Centre, The Cochrane Collaboration, 2021). Depending on the result category, the risk ratio (RR) and 95% confidence interval (CI) were used to calculate the categorical data, and the weighted mean difference (WMD) and 95% CI were used to calculate the continuous data. *p* < 0.05 was considered statistically significant. Heterogeneity was estimated by Cochran’s Q test and assessed using I^2^. When I2 > 50%, it is considered to have significant heterogeneity and a random-effects model is used to estimate the pooled effect. In contrast, a fixed-effects model was used. Besides, if there were heterogeneity in the results, we would look for heterogeneity sources and perform a sensitivity analysis by converting the random effect model and fixed effect model to determine whether the results are stable. Publication bias was analyzed visually through funnel plots.

Association rules of Chinese medicinals were analyzed by SPSS Modeler 18.0. Cytoscape V3.9.0 established the topological network.

## Results

### Study identification

**FIGURE 1:**
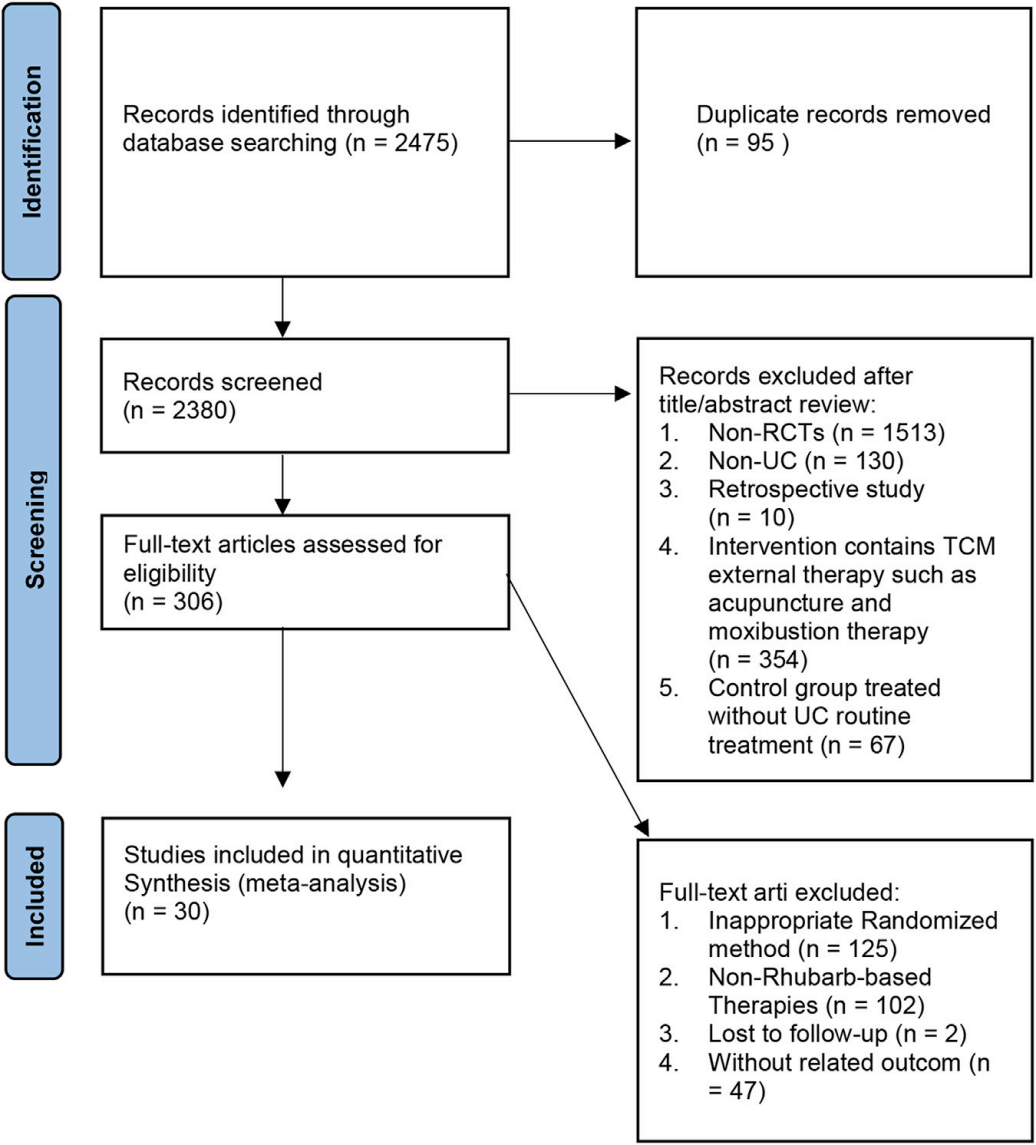
The flow of original literature selection.

Based on the search strategy, 2,475 potential associated articles were obtained by searching the databases. After deleting 95 repetitive articles, 2,380 articles were obtained. In the process of title and abstract screening, a total of 2,074 articles were excluded due to not meeting the inclusion criteria, and 306 papers were searched for further evaluation. Finally, 30 studies were finally retained for this Meta-analysis (Nong, 2009; Li, 2012; Deng, 2015; You et al., 2015; Ding, 2016; Wang, 2016; Zhang, 2016; Fei, 2017; Sheng et al., 2017; Shi and Sun, 2017; Tian, 2017; Wang et al., 2017; Wen and Sun, 2017; Yang et al., 2017; Wang, 2018; Guo and Ding, 2019; Li, 2019; Liu, 2019; Chen and Li, 2020; Deng and Chen, 2020; Sun and Zhang, 2020; Tan et al., 2020; Wang, 2020; Yuan, 2020; Zhang, 2020; Zhao, 2020; Li et al., 2021; Xue and Xu, 2021; Yang and Wang, 2021; Yin et al., 2021). Exclusive reasons are shown in Figure 1.

### Study characteristics

Supplementary Table S1 shows the general characteristics of 30 studies. A total of 2,507 patients were included from 2009 to 2020, with 1,257 cases in the treatment group and 1,250 cases in the control group. 5-ASA was selected as the control group measure in 20 studies, SASP in seven studies. The rhubarb-based medicinal formulas were used alone in the treatment group for four studies. And the rest are in combination with the UC routine treatment. All 30 studies were conducted in China.

### Risk of bias

**FIGURE 2:**
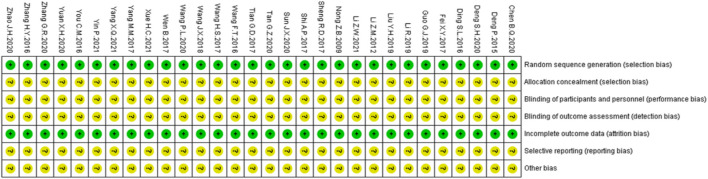
Results of the risk of bias assessment of the original literature included in the Meta-analysis.

Figure 2 shows the methodological quality of 30 studies. The risk of bias in the study was evaluated according to the criteria in the *Handbook for Systematic Evaluation of Cochrane Interventions* (Higgins et al., 2011).

All studies mentioned “randomness” and described the specific random assignment method for random sequence generation and were therefore judged as low risk. None of the studies described allocation concealment, blind of participants and personnel, or blind of outcomes, so the risks were unknown. Since the pre-registered protocols could not be acquired from the main authors, the selective report was rendered as unknown. Other risks of bias were also considered unclear.

#### Primary outcome

##### Clinical efficiency

**FIGURE 3:**
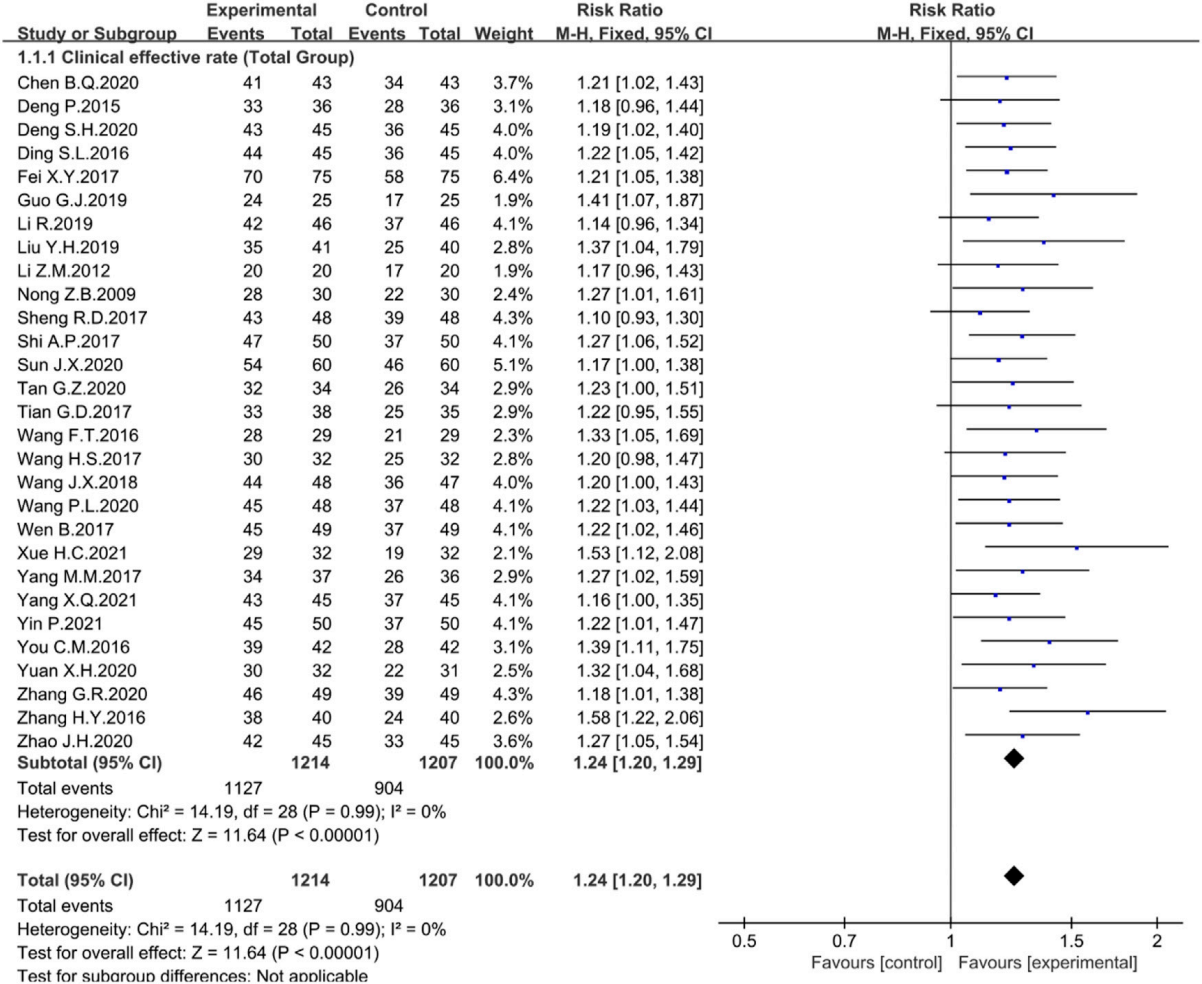
Forest plot displaying the Meta-analysis results for clinical efficiency.

The clinical efficiency was recorded in 29 trials. There was no heterogeneity among studies, and a fixed-effects model was used for analysis. The Meta-analysis results showed that the clinical efficiency of rhubarb-based treatment group was significantly higher than that of control group (*n* = 2421, I^2^ = 0%, RR = 1.24, 95% CI [1.20, 1.29], *p* < 0.00001) (Figure 3). Next, the subgroup analyses were performed.

To further analyze the effect of rhubarb-based medicinal formulas alone or combined with first-line drugs 5-ASA, SASP, or other different routine drugs in the experiment, the clinical efficiency was selected for subgroup analysis. There was no heterogeneity among the studies. A fixed-effect model was selected for Meta-analysis.

**FIGURE 4:**
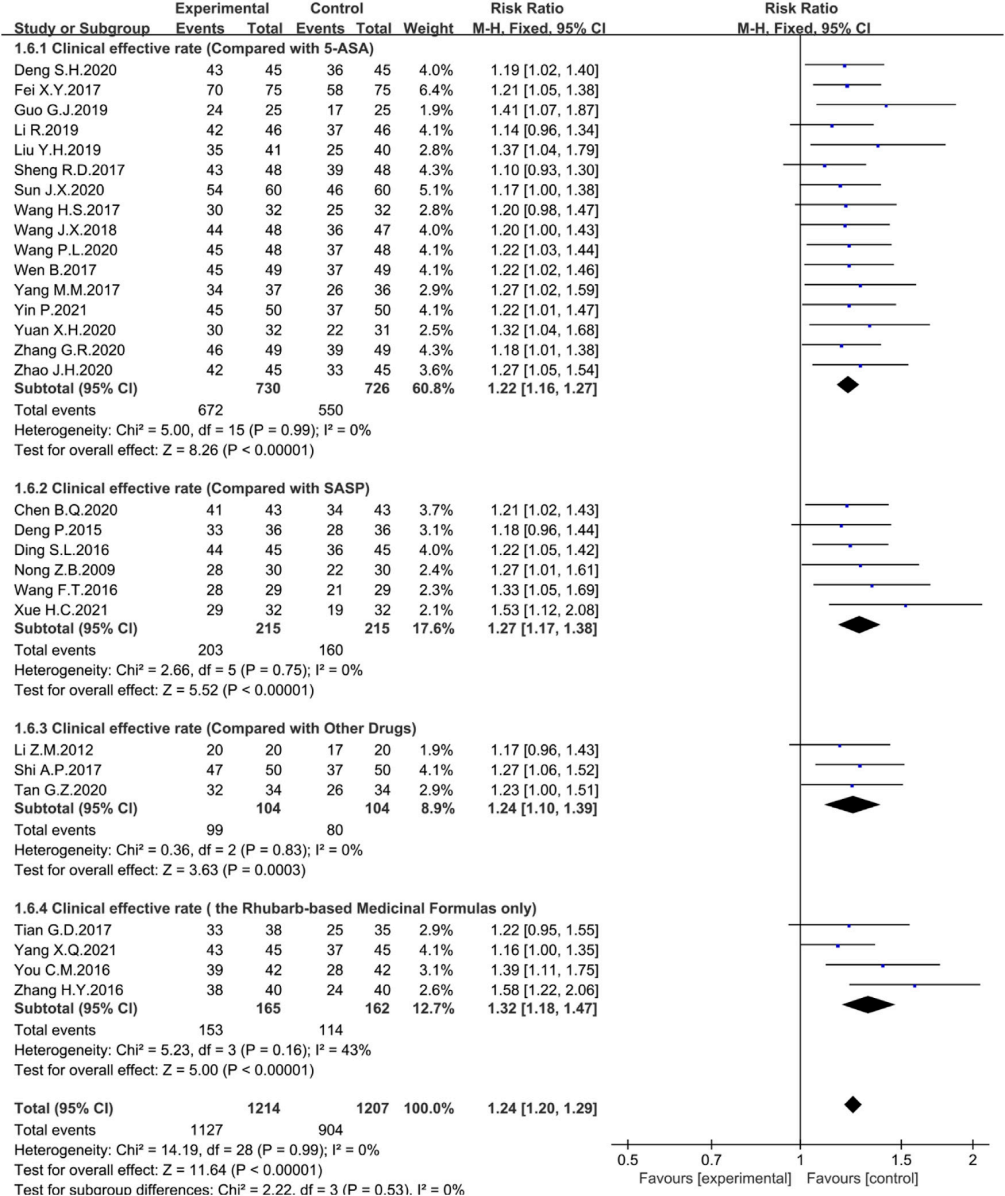
Forest plot displaying the results of the Meta-analysis for clinical efficiency in the rhubarb-based medicinal formulas alone or combined with other drugs.

In the treatment group, 16 studies combined with 5-ASA (*n* = 1456, I^2^ = 0%, RR = 1.22, 95% CI [ 1.16, 1.27], *p* < 0.00001) (Figure 4), six studies combined with SASP (*n* = 430, I^2^ = 0%, RR = 1.27, 95% CI [1.17, 1.38], *p* < 0.00001) (Figure 4), three studies combined with other routine drugs (*n* = 208, I^2^ = 0%, RR = 1.24, 95% CI [1.10, 1.39], *p* = 0.0003) (Figure 4), and four studies were conducted by the rhubarb-based medicinal formulas administered alone (*n* = 327, I^2^ = 43%, RR = 1.32, 95% CI [1.18, 1.47], *p* < 0.00001) (Figure 4). Compared with the control group, the clinical efficiency of the treatment group was significantly higher. The rhubarb-based medicinal formulas alone or combined with common drugs showed good therapeutic effects.

**FIGURE 5:**
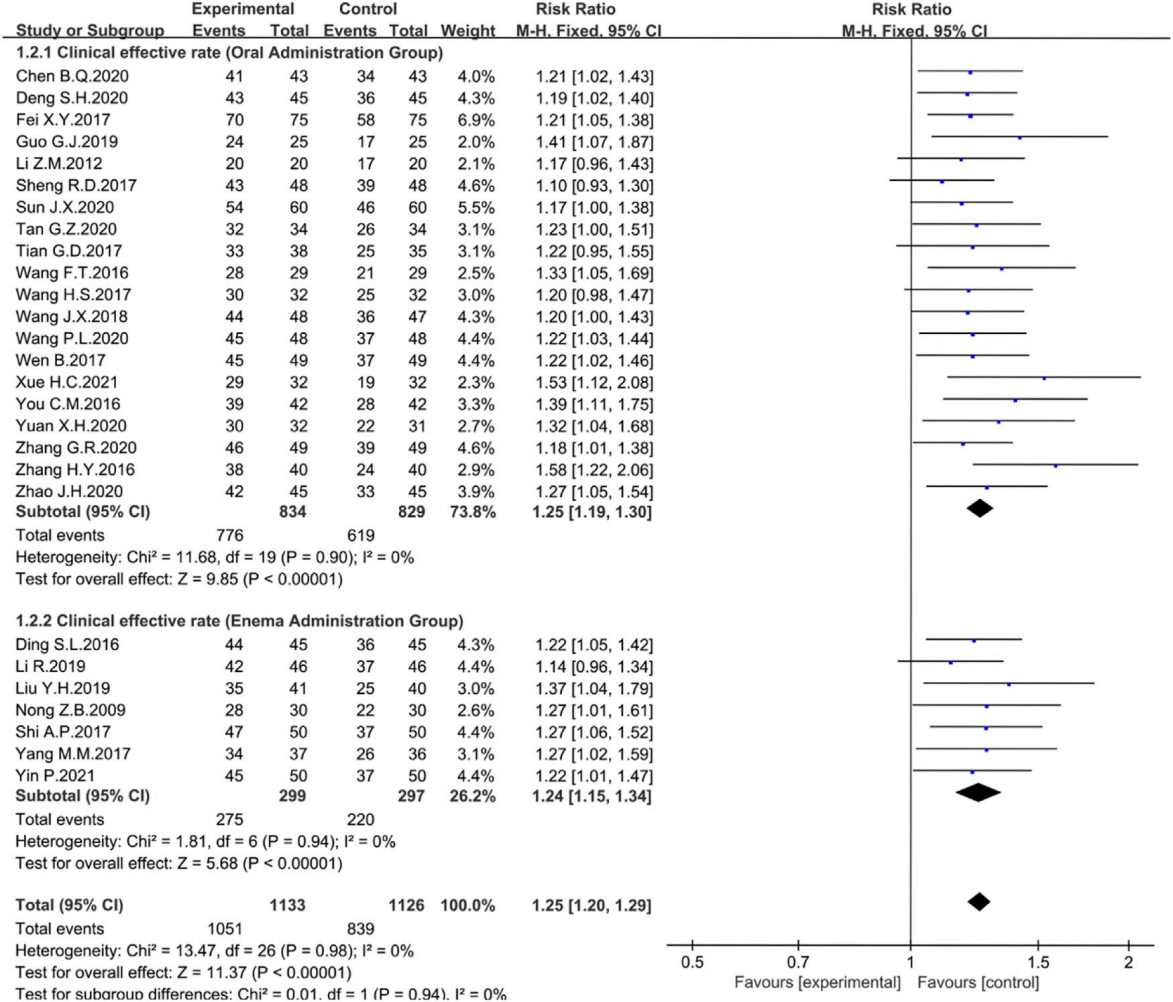
Forest plot displaying the results of the Meta-analysis for clinical efficiency in different dosing regimens.

Among the 29 studies, 21 were treated with rhubarb-based therapy oral administration and seven were treated with enema. Statistical analysis showed that no matter oral administration (*n* = 1318, I^2^ = 0%, RR = 1.25, 95% CI [1.19, 1.30], *p* < 0.00001) (Figure 5) or enema administration (*n* = 496, I^2^ = 0%, RR = 1.24, 95% CI [1.15, 1.34], *p* < 0.00001), the clinical efficiency was significantly improved (Figure 5).

**FIGURE 6:**
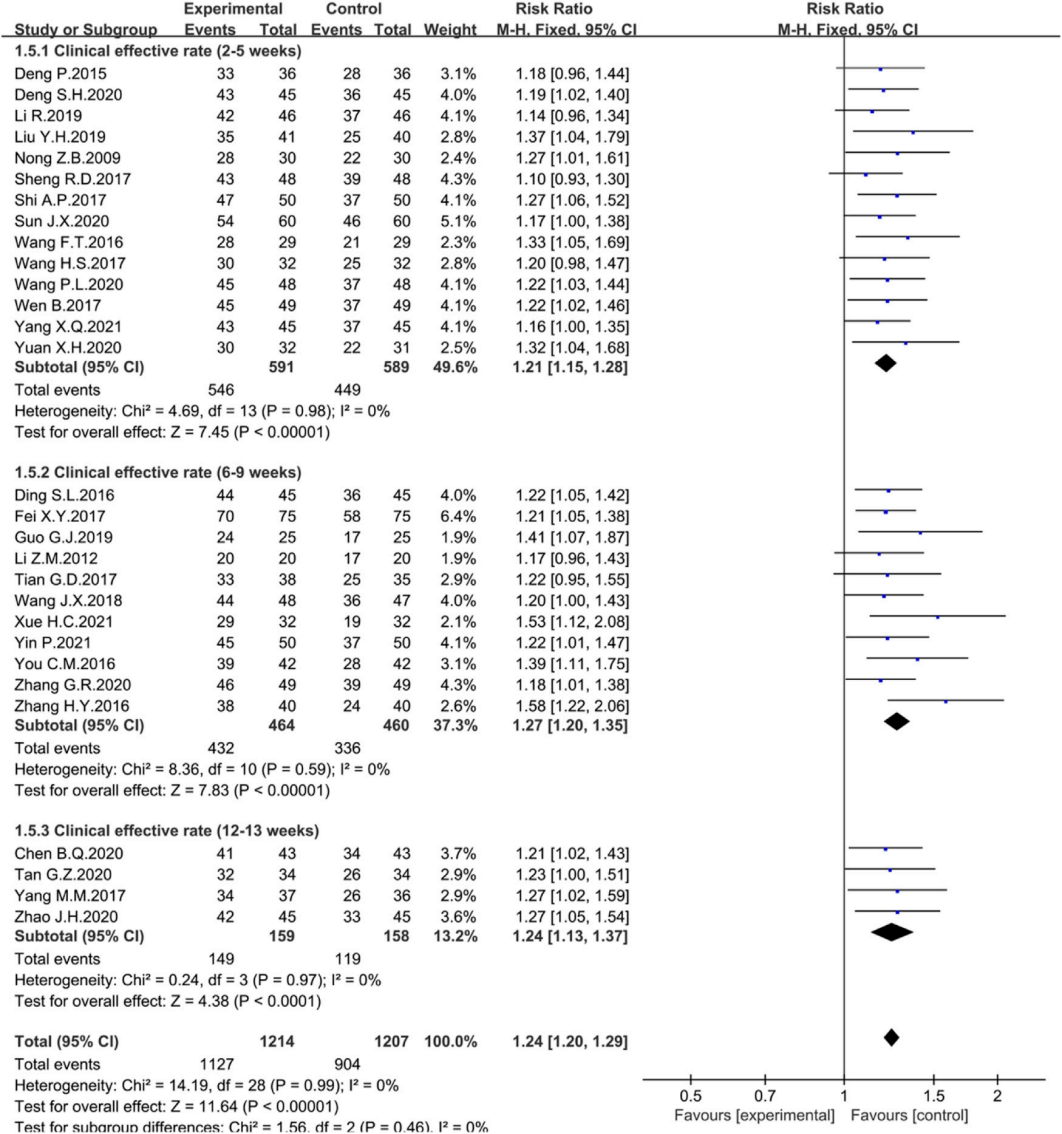
Forest plot displaying the results of the Meta-analysis for clinical efficiency in different courses of treatment.

The course of the treatments is one of the factors affecting the efficacy. In the studies where total effective rates were reported, treatment courses were divided into 2–5 weeks (*n* = 14); 6–9 weeks (*n* = 11) or 12–13 weeks (*n* = 4). None of these analyses showed heterogeneity, so the fixed effects model was chosen for the Meta-analysis. The results showed that the clinical efficiency significantly increased at 2–5 weeks (*n* = 1180, I^2^ = 0%, RR = 1.21, 95% CI [1.15, 1.28], *p* < 0.00001) (Figure 6), 6–9 weeks (*n* = 924, I^2^ = 0%, RR = 1.27, 95% CI [1.20, 1.35], *p* < 0.00001) (Figure 6), and 12–13 weeks (*n* = 317, I^2^ = 0%, RR = 1.24, 95% CI [1.13, 1.37], *p* < 0.0001) (Figure 6).

#### Secondary outcomes

##### Mayo score

**FIGURE 7:**
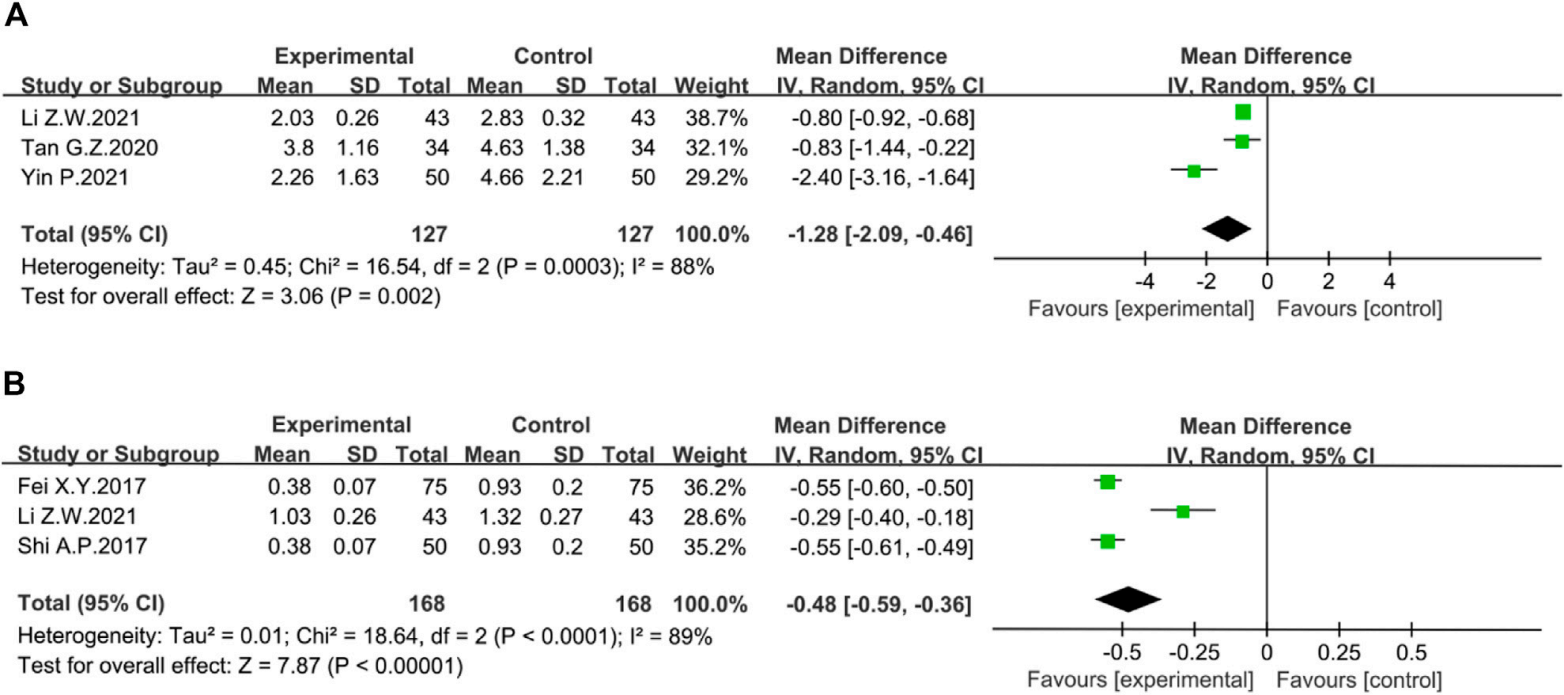
Forest plot showing the result of **(A)** Mayo score; **(B)** Geboes score.

Three trials reported Mayo score. The random-effect Meta-analysis showed that the Mayo score in the treatment group was significantly lower than that in the control group (*n* = 254, I^2^ = 88%; MD = −1.28; 95% CI [−2.09, −0.46], *p* = 0.002) (Figure 7). The sensitivity analysis showed that the study reported by Yin P. was the source of heterogeneity. Excluding this study reduced the inter-study heterogeneity (I^2^ = 0%).

##### Geboes score

The Geboes score was assessed in three studies. The heterogeneity among studies was obvious, so the random-effect model was used for Meta-analysis. A significant reduction in Geboes scores was seen in the treatment group compared to the control group. (*n* = 336, I^2^ = 89%; MD = −0.48; 95% CI [−0.59, −0.36], *p* < 0.00001) (Figure 7). According to sensitivity analysis, the source of heterogeneity was reported by Li Z.W. After excluding this study, heterogeneity was reduced to I^2^ = 0%.

##### Recurrence rate

**FIGURE 8:**
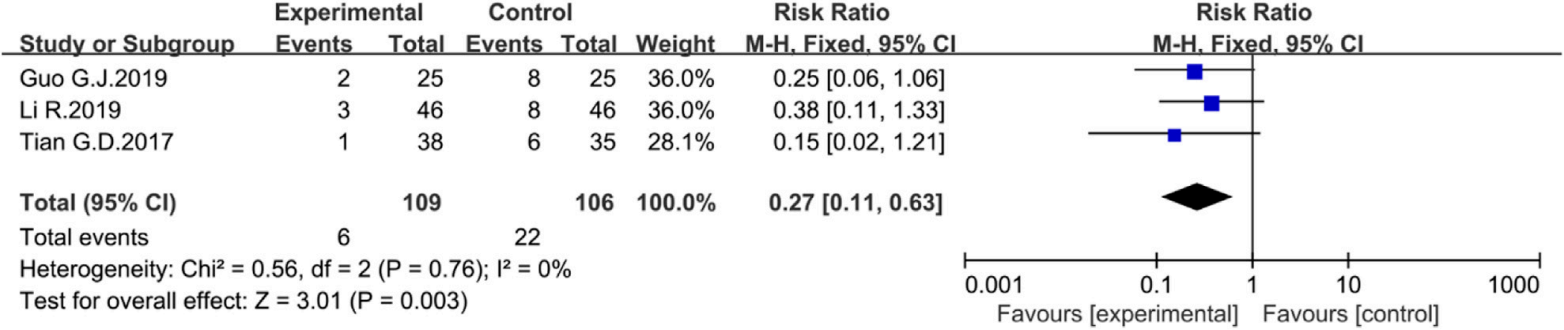
Forest plot displaying the results of the Meta-analysis for recurrence rate.

Three studies evaluated the reduction in recurrence rate. There was no heterogeneity among studies. The fixed-effects model was used in the analysis. The results revealed that the rhubarb-based treatment could significantly reduce the recurrence rate of UC patients compared with the control group (*n* = 215, I^2^ = 0%. RR = 0.27; 95% CI [0.11, 0.63], *p* = 0.003) (Figure 8).

##### PLT

**FIGURE 9:**
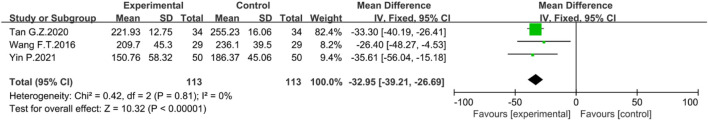
Forest plot showing the result of PLT.

PLT increase was evaluated in three trials. There was no heterogeneity among the studies, and the fixed-effects model was used in the analysis. The results showed that the rhubarb-based treatment could reduce PLT levels compared with the control group (*n* = 226, I^2^ = 0%. RR = −32.95; 95% CI [−39.21, −26.69], *p* < 0.00001) (Figure 9). Furthermore, it could also influence the coagulation-related indexes of prothrombin time (PT) and fibrinogen (FIB) (Supplementary Figure S1).

##### Inflammatory cytokines and protein

**FIGURE 10:**
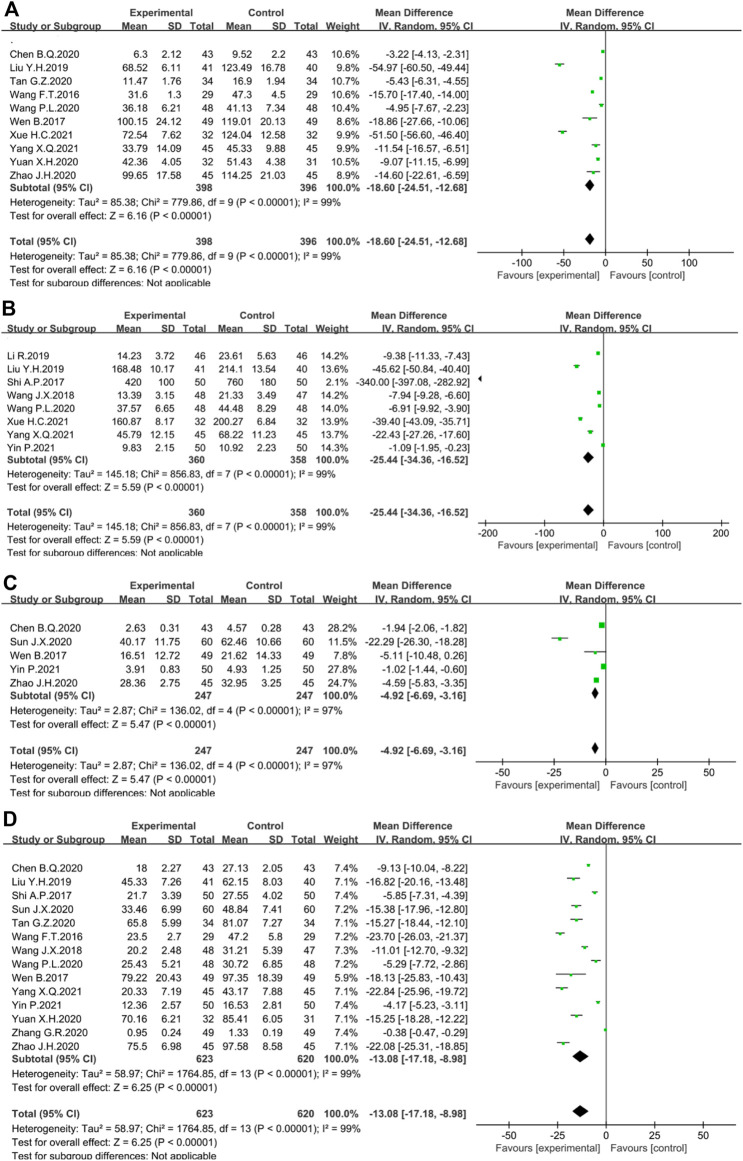
Forest plot displaying the results of the Meta-analysis for inflammation-related cytokines and protein. The results of **(A)** IL-6; **(B)** IL-8; **(C)** IL-1β; **(D)** TNF-α; **(E)** CRP; **(F)** IL-10.

**FIGURE 11:**
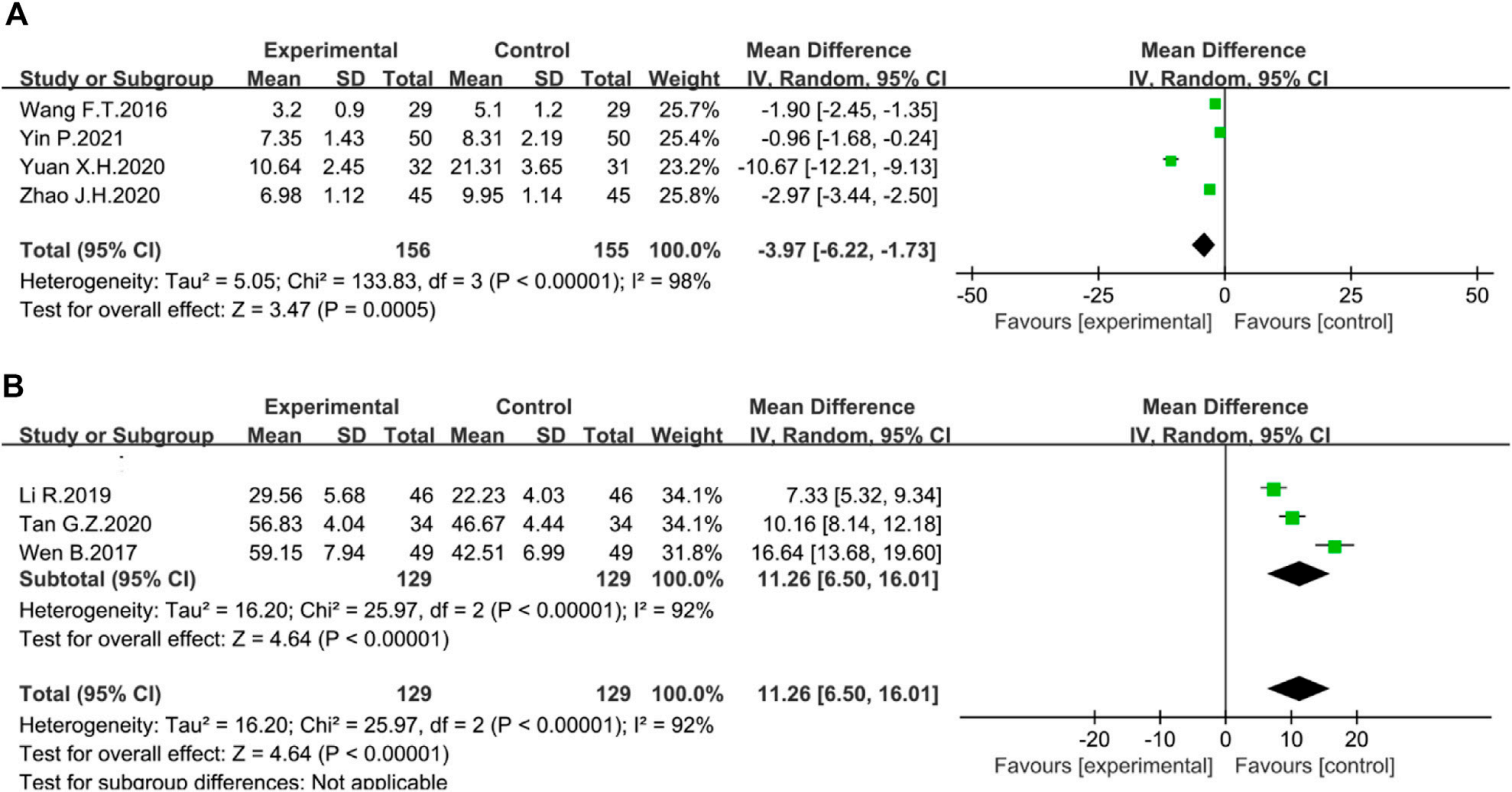
Forest plot displaying the results of the Meta-analysis for inflammation-related cytokines and protein. The results of **(A)** CRP; **(B)** IL-10.

The regulation of cytokines towards multiple inflammatory signaling pathways are the central component of chronic inflammation in UC. IL-6, one of the major cytokines, can bind to IL-6R to activate STAT3 signaling and further induce differentiation of Th17 cells, leading to excessive release of pro-inflammatory cytokines such as IL-17 and IL22 (Waldner and Neurath, 2014). IL-6 also stimulates the production of the regulatory inflammatory marker C-reactive protein (CRP) (Pepys and Hirschfield, 2003). Meanwhile, the synergy effect of IL-1β, IL-23, and IL-6 strengthens Th17 cell differentiation and maintains the expression of pro-inflammatory cytokines (Chung et al., 2009). Moreover, IL-6 and IL-8, as inducers of neutrophil recruitment, can amplify the inflammatory response by interacting with a large number of inflammatory cells exuding from inflamed areas (Kim and Kim, 2010; Waldner and Neurath, 2014). TNF-α stimulates death receptor signaling leads to excessive crypt cell death, which increases the intestinal inflammatory response (Günther et al., 2011). The results showed that the rhubarb-based therapy significantly reduced the level of IL-6 (*n* = 794, I^2^ = 99%. MD = −18.60; 95% CI [−24.51, −12.68], *p* < 0.00001) (Figure 10A), IL-8 (*n* = 718, I^2^ = 99%. MD = −25.44; 95% CI [−34.36, −16.52], *p* < 0.00001) (Figure 10B), IL-1β (*n* = 494, I^2^ = 97%. MD = −4.92; 95% CI [−6.69, −3.16], *p* < 0.00001) (Figure 10C), TNF-α (n = 1243, I^2^ = 99%. MD = −13.08; 95% CI [−17.18, −8.98], *p* < 0.00001) (Figure 10D), and CRP (n = 311, I^2^ = 98%. MD = −3.97; 95% CI [−6.22, −1.73], *p* = 0.0005) (Figure 11A) when compared with the control group. Overall, IL-6, IL-8, IL-1β, and TNF-α are all considered to be essential mediators of chronic intestinal inflammation, and the rhubarb-based treatment showed a better effect on pro-inflammatory cytokines.

Besides, the relative deficiency of IL-10 in UC patients may contribute to persistent inflammatory changes (Wang et al., 2020). IL-10 down-regulates the production of Th1-derived cytokines to exert anti-inflammatory effects (Glocker et al., 2011). The pooled analysis suggested that the difference between the two groups was statistically significant (*n* = 258, I^2^ = 92%. MD = 11.26; 95% CI [6.50, 16.01], *p* < 0.00001) (Figure 11B).

##### Adverse events

**FIGURE 12:**
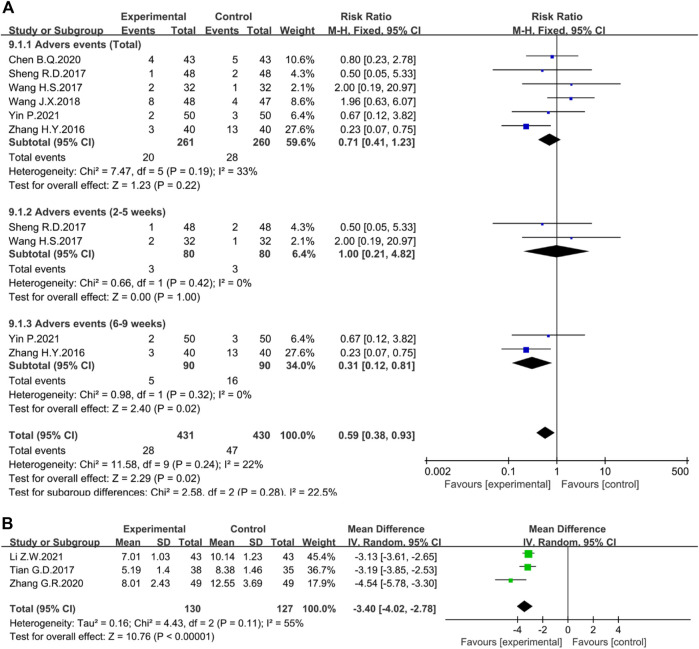
Forest plot displaying the results of the Meta-analysis for **(A)** adverse events and **(B)** the TCM symptoms integral.

Six studies reported adverse reactions. Nevertheless, the results were not statistically significant (*n* = 521, I^2^ = 33%, RR = 0.71, 95% CI [0.41, 1.23], *p* = 0.22) (Figure 12A). Due to the different courses of treatment, another subgroup analysis was set up. After 6–9 weeks of administration, rhubarb-based therapy had an obvious effect on reducing the incidence of adverse events (*n* = 180, I^2^ = 0%, RR = 0.31, 95% CI [0.12, 0.81], *p* = 0.02) (Figure 12A). However, the course of 2–5 weeks (*n* = 160, I^2^ = 0%, RR = 1.00, 95% CI [0.21, 4.82], *p* = 1.00) (Figure 12A) did not show statistical significance.

##### Symptoms integral

The TCM symptoms integral mainly includes the total symptom score, individual symptom scores such as abdominal pain, diarrhea, urgency, pus and blood stool, and burning pain in the anus. The total symptom score mainly observes symptoms such as diarrhea, abdominal pain, and pus and blood stool, from mild to severe, and scores 0, 1, 2, and 3, respectively. The higher the score, the more serious the symptoms. Three studies evaluated the total symptom score. The random-effect Meta-analysis suggested that the difference between the two groups was statistically significant (*n* = 257, I^2^ = 55%; MD = −3.40; 95% CI [−4.02, −2.78], *p* < 0.00001) (Figure 12B). The sensitivity analysis showed that the study reported by Zhang G.R was the source of heterogeneity because excluding this study resulted in an I^2^ = 0%.

Furthermore, the investigation also evaluated the individual symptom scores of abdominal pain, diarrhea, urgency, pus and blood stool, and burning anal pain. Although the heterogeneity was high, it indicated that rhubarb-based therapy could improve UC symptoms to some extent (Supplementary Figures S2, S3).

##### Publication bias

Publication bias was assessed by funnel plots for the included 29 studies which reported the primary outcome. The funnel plots were asymmetrical, suggesting a possible publication bias (Supplementary Figure S4).

## Discussion

UC is one of the modern intractable diseases regarded by the World Health Organization (Pedersen et al., 2014). Longstanding UC bears a high risk of developing colitis-associated colon cancer (Ekbom et al., 1990). Recently, 5-ASA is the most widely used first-line drug which is obviously beneficial to mild to moderate UC but limited for severe UC (Ko et al., 2019). As the supplementary drugs, corticosteroids and immunosuppressive drugs often required by the severe patients. However, the long-term application of corticosteroids and immunosuppressive drugs always brings about significant side effects (Ko et al., 2019; Kobayashi et al., 2020). What’s more, surgery, as an optional therapy for sever UC, is mentioned to delay the progression of the disease, but the prognosis of surgery still carries the risk of colon cancer (Windsor et al., 2013). Basically, TCM has attracted increasing attention for the potential effective and safe treatment.

TFTM is a typical therapeutic method in TCM. According to TCM theory, TFTM mainly refers to those medicinals with purgative method. There are many representative medicinals of TFTM, such as *Citrus × aurantium* L. [Rutaceae; *Citrus × aurantium* young fruit], *Magnolia officinalis* Rehder & E.H.Wilson [Magnoliaceae; *Magnolia officinalis* bark], *Aloe vera* (L.) Burm.f. [Asphodelaceae; *Aloe vera* sap], *Cannabis sativa* L. [Cannabaceae; *Cannabis sativa* ripe fruit], which have been shown to treat experimental UC mainly by anti-inflammation and modulating intestinal flora (He et al., 2018; Naini et al., 2021; Eom et al., 2022; Xie et al., 2022). But compared with those medicinals, rhubarb is the most representative, and it is also the most widely used in the treatment of gastrointestinal diseases (Zhang et al., 2021). Rhubarb has been validated to treat UC through immunosuppression, anti-inflammation, intestinal flora regulation, and endotoxin inhibition (Miao et al., 2018; Xiang et al., 2020). The potential clinical value is necessary to be assessed with evidence-based methods.

In this study, 30 RCTs involving 2,421 patients were included in the Meta-analysis. The results reveal that the clinical efficiency of the rhubarb-based treatment group was significantly higher than that of the control group. Furthermore, a comparable number of RCTs evaluated the use of rhubarb-based medicinal formulas alone, supporting its effectiveness explicitly. 5-ASA and SASP belong to the 5-ASA class of medications, both of whom are the mainstay of treatment of mild to moderate UC (Ko et al., 2019). Long-term use of 5-ASA reduces the risk of relapse and cancer in UC patients, making it an indispensable drug for UC treatment (Kim, 2018; Wang et al., 2018). Nevertheless, for patients with severe UC, the therapeutic effect of 5-ASA is limited. Of note, the rhubarb-based medicinal formulas combined with 5-ASA or SASP were more effective than the use of 5-ASA or SASP alone, indicating that the synergistic effect of rhubarb is remarkable. The result suggests that rhubarb-based therapy might benefit patients who did not respond or could not tolerate 5-ASA treatment, thus avoiding the apparent side effects associated with the use of corticosteroid and thiopurine.

Further, patients with proctitis are always treated with 5-ASA suppositories, which target the site of inflammation directly and seem to be more effective than oral 5-ASA. In left-sided colitis, 5-ASA should be administered as an enema instead of a suppository in order to reach the splenic flexure (Gionchetti et al., 1998; Choi et al., 2015; Reinisch et al., 2015). As with 5-ASA, different administration routes of medicinals can directly impact the efficiency rate. This study found that the rhubarb-based therapy was clinically more effective when administered either orally or by enema. What’s more, it was meaningful to add it to any course of treatment.

The symptoms of UC mainly include abdominal pain, vomiting, diarrhea, and rectal bleeding, which seriously affect life quality of patients. Studies have shown that rhubarb could exert various pharmacological effects in the gastrointestinal tract (Xiang et al., 2020). The combination of anthraquinones in rhubarb played a role in inducing diarrhea, while tannic acid could inhibit the purgative effect of anthraquinones and thereby induced antidiarrheal effects (Qin et al., 2011; Cao et al., 2017). Through the review of TCM symptoms integral, it was found that rhubarb-based therapy could significantly improve the symptoms of abdominal pain, diarrhea, urgency, pus and blood stool, and burning anal pain in UC patients, and ultimately had a positive impact on the symptomology and quality of life of patients. Also, Mayo and Geboes scores revealed that the rhubarb-based therapy effectively improved the disease activity and the histological status of UC. And the result of the recurrence rate indicated that the disease process was effectively controlled and recurrence was delayed.

The hypercoagulable state is an essential part of the pathological mechanism of UC (Danese et al., 2007). Meanwhile, cytokines regulated by multiple inflammatory signaling pathways are a central component of chronic inflammation (Ungaro et al., 2017; Kobayashi et al., 2020). The imbalance of cytokines have been reported in patients with active IBD, including TNF-α, IL-1β, IL-6, IL-8, IL-10, IL-12, IL-17, IL-23, and TGF-β (Neurath, 2014). The simultaneous activation of coagulation and inflammation in colon injury foci are identified as the preservation mechanisms for repairing damaged areas (Lipinski et al., 2011). At the same time, the crosstalk between coagulation and inflammation has raised concerns. Inflammatory stimuli can trigger the coagulation cascade while coagulation also regulates inflammatory signaling pathways (de Maat et al., 2014). PLT, a principal coagulation marker, constitutes a crucial link between inflammation and coagulation, creating a vicious circle in which participating parameters conserve and propagate each other (Yoshida and Granger, 2009). PLT is activated at inflammatory sites and excrete large amounts of pro-inflammatory substances located in its intracellular granules (Smyth et al., 2009). The results of this Meta-analysis showed that rhubarb-based therapy significantly reduced PLT, TNF-α, IL-1β, IL-6, IL-1β, and CRP, and increased IL-10, as well as had some effects on PT and FIB. Thus, rhubarb-based therapy can improve the hypercoagulable state of UC and suppress intestinal inflammation. An important point to note was the highly heterogeneous results in the analysis of all six inflammatory cytokines, likely caused by the use of different measurement devices and measurement units in the original study. The results of this Meta-analysis should be interpreted with caution.

Moreover, rhubarb has a powerful blood-activating effect. It is a common medicinal for blood stasis syndrome (Liu et al., 2013; Gao et al., 2020). Studies have reported that rhubarb had significant anti-PLT effects and PLTs were the main effector cells of rhubarb in the promoting blood circulation to remove blood stasis method (Seo et al., 2012; Gao et al., 2020). It could significantly improve whole blood viscosity and plasma viscosity in high molecular dextran-induced hyperviscosity syndrome model in rats, and reduce the levels of PLT activation markers Plasma P-selectin and thromboxane (Gao et al., 2020). Additionally, rhubarb significantly affected local microcirculation, reducing local pancreatic blood flow in the pancreas and improving pancreatic microcirculation (Zhao et al., 2004). It protected the intestinal mucosal capillary endothelial cells and increased the number of functional capillaries, promoted blood flow through intestinal mucosal capillaries, reduced thrombogenesis, and improved the blood and oxygen supply of the intestinal mucosa (Cui et al., 2014; Cui et al., 2016). Derivatives of rhubarb also had anti-PLT aggregation effects, among which, chrysophanol-8-O-glucoside, an anthraquinone derivative isolated from rhubarb, was found to have the most potent inhibitory effect on collagen- and thrombin-induced PLT aggregation and was mightier than the positive drug aspirin. Specifically, it was found to inhibit PLT aggregation *in vitro* and *ex vivo* and prolong bleeding time and activate partial thromboplastin time (Seo et al., 2012). In addition, the mixture of five monomeric compounds of rhubarb could antagonize the matrix Metalloproteinase-9-induced human umbilical vein endothelial cell (HUVEC) monolayer permeability by promoting HUVEC proliferation and reducing extracellular vascular endothelial-cadherin concentrations (Cui et al., 2016). In addition, although the bioavailability of rhubarb and its monomers may be low, it seems that we can consider combining them with nanosystems (Zhang et al., 2022a; Zhang et al., 2022b). In conclusion, based on the characteristics of coagulation abnormalities associated with UC and the exact clinical efficacy and experimental evidence of rhubarb in UC, the rhubarb’s coagulation-regulation effect is worthy of further investigation.

The incidence of adverse events is an important indicator to evaluate the safety of a treatment strategy. Several studies have reported that 5-ASA and SASP may cause adverse events such as fever, nausea, dizziness, and diarrhea (Sehgal et al., 2018). In five of the six studies that reported adverse events, the control drug was 5-ASA class. Although a number of studies have reported that medicinals could reduce the incidence of adverse time when used in combination with 5-ASA (Chen et al., 2020), the results showed that rhubarb-based therapy did not significantly improve these side effects. For the subgroup analysis, the incidence of side effects was not statistically different at 2–5 weeks of treatment while showed a significant difference at 6–9 weeks. In addition, although rhubarb has the side effect of purgative, the results of our analysis showed that rhubarb-based therapy did not exhibit significant side effects. This means it has a high safety profile in clinical use. This article concludes that the rhubarb-based therapy can improve clinical efficacy, improve clinical symptoms, and reduce recurrence rate in UC. It can also meliorate the hypercoagulable and inflammatory status. In addition, subgroup analysis suggests that early intervention of rhubarb-based therapy will obtain better effects. There is a synergistic effect of the rhubarb-based medicinal formulas with 5-ASA or SASP. Nevertheless, the mechanism of the synergistic effect has not been revealed completely that require more investigations.

**FIGURE 13:**
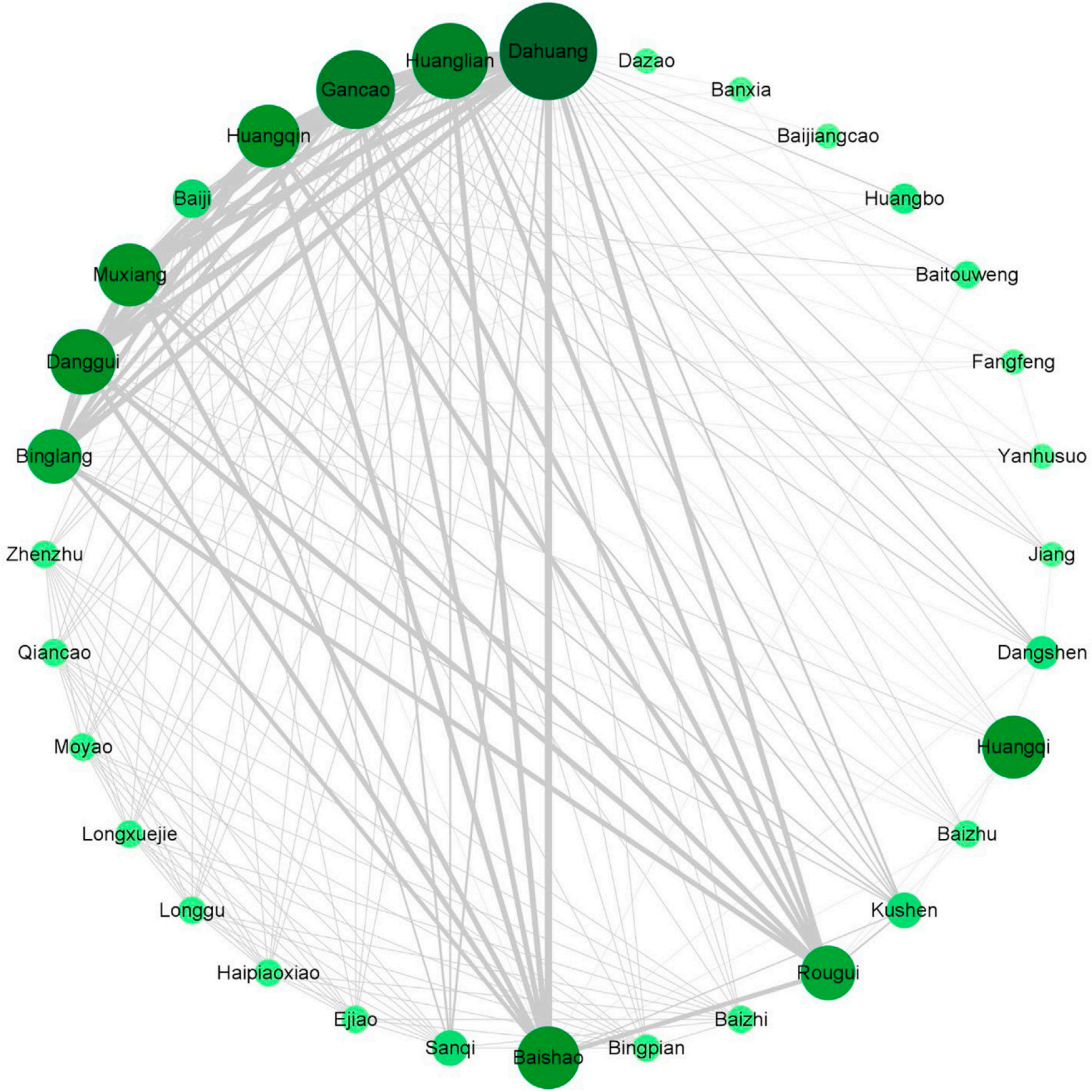
A complex network analysis of the rhubarb-based medicinal formulas. The size and color of the nodes represent the frequency of occurrence of the medicinals. The larger and darker the node, the more frequently the medicinals appear. The degree of association between medicinals is marked by lines, the thicker the line and the darker the color, the stronger the association between two botanical drugs.

To determine the characteristics and patterns of specific medicinals used in the rhubarb-based medicinal formulas, this investigation analyzed the medicinals used in each original study (Supplementary Figure S5). The high-frequency medicinals (frequency ≥14) were rhubarb, *Glycyrrhiza glabra* L. [*gān căo*; Fabaceae; *Glycyrrhiza glabra* root and rhizome], *Coptis chinensis* Franch. [*huáng lián*; Ranunculaceae; *Coptis chinensis* rhizome], *Scutellaria baicalensis* Georgi [*huáng qín*; Lamiaceae; *Scutellaria baicalensis* root], *Angelica sinensis*(Oliv.)Diels [*dāng guī*; Apiaceae; *Angelica sinensis* root], *Paeonia lactiflora* Pall. [*bái sháo*; Paeoniaceae; *Paeonia lactiflora* root], and *Dolomiaea costus* (Falc.) Kasana & A.K.Pandey [*mù xiāng*; Asteraceae; *Dolomiaea costus* root], *Areca catechu* L. [*bīng láng*; Arecaceae; *Areca catechu* seed], and *Neolitsea cassia* (L.) Kosterm. [*ròu guì*; Lauraceae; *Neolitsea cassia* bark], with frequencies of 30, 23, 22, 20, 18, 17, 17, 15, 14, respectively. In order to determine the characteristics and patterns of specific medicinals used in the included studies, an association analysis was performed (Figure 13). According to the frequency, correlation, and confidence, *dà huáng*, *huáng lián*, and *huáng qín* formed the major group of medicinals that make up the classic formula Xiexin decoction (XXD). XXD has been recorded since the Han Dynasty (early third century), in *Essentials from the Golden Cabinet* (*Jīn Guì Yào Lüè*), which has been commonly used to treat patients with chronic gastritis, peptic ulcer, acute dysentery, UC, or other dysfunctions of the gastrointestinal tract in the clinic. According to the TCM, *dà huáng*, *huáng lián*, and *huáng qín* are all heat-clearing and damp-drying medicinals (Teng et al., 2019). And the dampness syndrome and large intestine damp-heat syndrome are the most common syndrome of UC patients (Zhang et al., 2019). XXD can clear heat and dry dampness, which is often applied to treat UC patients effectively (Jia et al., 2021). Research has shown that XXD could promote the recovery of colitis and inhibit the colonic inflammation damage in UC rats by reducing the level of MPO and the expression of TNF-α and NF-κB, and increasing the production of IL-10 in colon tissues (Han et al., 2013). The pattern analysis provides a more precise decoction strategy for the clinical application of rhubarb-based medicinal formulas, which raises an optional protocol for UC patients.

## Limitation

As the available studies only provided short-term assessments of 2–13 weeks, this investigation is not able to assess the long-term efficacy and safety of the rhubarb-based therapy for UC. Second, the lack of a large number of high-quality, multicenter standard RCTs and the lack of description of random assignment procedures and blinding in some studies may also affectthe quality of this Meta-analysis. Furthermore, even though this paper used sensitivity analysis to reduce the effect of different measurement units and methods on the results, the outcomes of inflammatory cytokines, and TCM symptoms integral to this Meta-analysis still showed a high level of heterogeneity. Hence, the results of the analysis should be identified carefully. It would be beneficial if more research would be conducted to promote the rational application of rhubarb-based therapy for UC. In addition, the other representative medicinals of TFTM are widely used in clinical practice, but there are few studies related to UC, and their recognition is not as high as rhubarb in the TCM industry. This makes them not the object of our Meta-analysis. But their value also deserves more exploration in the future.

## Conclusion

In this article, rhubarb-based therapy is effective in improving UC, especially in terms of relieving clinical symptoms, reducing recurrences, and improving hypercoagulable states and inflammation. Furthermore, the rhubarb-based medicinal formulas combined with 5-ASA or SASP are more effective than 5-ASA or SASP alone. And rhubarb-based therapy is recommend use within 1–13 weeks or 3 months *via* administered orally or by enema, which is contributes to ensure the curative effect and avoid its side effects. As an important case of TFTM, rhubarb-based therapy provides evidence for the practical application of TFTM, and also provides ideas for further development and exploration.

## Data Availability

The original contributions presented in the study are included in the article/Supplementary Material, further inquiries can be directed to the corresponding authors.

## Abbreviations

CBM, Chinese Scientific Journal Database; CNKI, Chinese National Knowledge Infrastructure; CRP, C-reaction protein; EMBASE, Excerpta Medica Database; FIB, fibrinogen; IBD, inflammatory bowel disease; IL-6, interleukin-6; PLT, platelet; PT, prothrombin time; RCTs, Randomized clinical trials; TCM, traditional Chinese medicine; TFTM, Tong-fu therapeutic method; TNF-α, tumor necrosis factor-α; UC, Ulcerative colitis; XXD, Xiexin decoction.

## References

[B1] Cao Y. J. Pu Z. J. Tang Y. P. Shen J. Chen Y. Y. Kang A. (2017). Advances in bio-active constituents, pharmacology and clinical applications of rhubarb. Chin. Med. 12, 36. 10.1186/s13020-017-0158-5 29299052 PMC5745730

[B2] Chen B. Q. Li Y. (2020). Effect of dachaihu decoction combined with sulfasalazine enteric-coated tablets in the treatment of ulcerative colitis. Med. Innovation China 17 (15). 10.3969/j.issn.1674-4985.2020.15.017

[B3] Chen M. Ding Y. Tong Z. (2020). Efficacy and safety of Sophora flavescens (kushen) based traditional Chinese medicine in the treatment of ulcerative colitis: Clinical evidence and potential mechanisms. Front. Pharmacol. 11, 603476. 10.3389/fphar.2020.603476 33362558 PMC7758483

[B4] Choi C. H. Rutter M. D. Askari A. Lee G. H. Warusavitarne J. Moorghen M. (2015). Forty-year analysis of colonoscopic surveillance program for neoplasia in ulcerative colitis: An updated overview. Am. J. Gastroenterol. 110 (7), 1022–1034. 10.1038/ajg.2015.65 25823771 PMC4517513

[B5] Chung Y. Chang S. H. Martinez G. J. Yang X. O. Nurieva R. Kang H. S. (2009). Critical regulation of early Th17 cell differentiation by interleukin-1 signaling. Immunity 30 (4), 576–587. 10.1016/j.immuni.2009.02.007 19362022 PMC2705871

[B6] Cui Y. L. Wang L. Tian Z. T. Lin Z. F. Chen D. C. (2014). Effect of rhubarb pre-treatment on intestinal microcirculation in septic rats. Am. J. Chin. Med. 42 (5), 1215–1227. 10.1142/s0192415x14500761 25176604

[B7] Cui Y. L. Zhang S. Tian Z. T. Lin Z. F. Chen D. C. (2016). Rhubarb antagonizes matrix metalloproteinase-9-induced vascular endothelial permeability. Chin. Med. J. 129 (14), 1737–1743. 10.4103/0366-6999.185859 27411464 PMC4960966

[B8] Danese S. Papa A. Saibeni S. Repici A. Malesci A. Vecchi M. (2007). Inflammation and coagulation in inflammatory bowel disease: The clot thickens. Am. J. Gastroenterol. 102 (1), 174–186. 10.1111/j.1572-0241.2006.00943.x 17100967

[B9] de Maat S. Tersteeg C. Herczenik E. Maas C. (2014). Tracking down contact activation - from coagulation in vitro to inflammation in vivo. Int. J. Lab. Hematol. 36 (3), 374–381. 10.1111/ijlh.12222 24750684

[B10] Deng P. (2015). 36 cases of ulcerative colitis treated with compound Qinbo granules orally combined with retention enema. Hunan J. TCM 31 (4), 54–55. 10.16808/j.cnki.issn1003-7705.2015.04.026

[B11] Deng S. H. Chen S. (2020). Efficacy observation of Zhikang capsule combined with mesalazine in the treatment of mild to moderate ulcerative colitis. Electron. J. Clin. Med. Literature 7 (34), 51–52. 10.16281/j.cnki.jocml.2020.34.037

[B12] Ding S. L. (2016). Clinical observation on the treatment of ulcerative colitis with large intestine damp-heat syndrome with qingrechangyufang enema. J. New Chin. Med. 48 (4). 10.13457/j.cnki.jncm.2016.04.086

[B13] Ekbom A. Helmick C. Zack M. Adami H. O. (1990). Increased risk of large-bowel cancer in Crohn's disease with colonic involvement. Lancet 336 (8711), 357–359. 10.1016/0140-6736(90)91889-i 1975343

[B14] Eom J. Y. Choi S. H. Kim H. J. Kim D. H. Bae J. H. Kwon G. S. (2022). Hemp-derived nanovesicles protect leaky gut and liver injury in dextran sodium sulfate-induced colitis. Int. J. Mol. Sci. 23 (17), 9955. 10.3390/ijms23179955 36077356 PMC9456148

[B15] Fei X. Y. (2017). Efficacy of Lishi Hexue Decoction assisted by mesalazine in the treatment of ulcerative colitis and its effect on quality of life and levels of inflammatory immune cytokines. J. Emerg. Traditional Chin. Med. 26 (6), 1097–1098. 10.3969/j.issn.1004-745X.2017.06.053

[B16] Gao D. Wu S. N. Zhang C. E. Li R. S. Liu Z. J. Xiao X. H. (2020). Exploration in the mechanism of rhubarb for the treatment of hyperviscosity syndrome based on network pharmacology. J. Ethnopharmacol. 261, 113078. 10.1016/j.jep.2020.113078 32534118

[B17] Gionchetti P. Rizzello F. Venturi A. Ferretti M. Brignola C. Miglioli M. (1998). Comparison of oral with rectal mesalazine in the treatment of ulcerative proctitis. Dis. Colon Rectum 41 (1), 93–97. 10.1007/bf02236902 9510317

[B18] Glocker E. O. Kotlarz D. Klein C. Shah N. Grimbacher B. (2011). IL-10 and IL-10 receptor defects in humans. Ann. N. Y. Acad. Sci. 1246, 102–107. 10.1111/j.1749-6632.2011.06339.x 22236434

[B19] Günther C. Martini E. Wittkopf N. Amann K. Weigmann B. Neumann H. (2011). Caspase-8 regulates TNF-α-induced epithelial necroptosis and terminal ileitis. Nature 477 (7364), 335–339. 10.1038/nature10400 21921917 PMC3373730

[B20] Guo G. J. Ding Q. X. (2019). Clinical observation of Tiaochangjiening decoction combined with Western medicine in the treatment of intestinal damp-heat ulcerative colitis. China's Naturop. 27 (10), 66–67. 10.19621/j.cnki.11-3555/r.2019.1035

[B21] Han X. H. Zhong J. Guo J. Y. Shi R. Wang X. H. Wang C. H. (2013). Relationships between pharmacokinetics and efficacy of Xie-xin decoction in rats with experimental ulcerative colitis. J. Ethnopharmacol. 148 (1), 182–189. 10.1016/j.jep.2013.04.008 23619018

[B22] He W. Li Y. Liu M. Yu H. Chen Q. Chen Y. (2018). Citrus aurantium L. and its flavonoids regulate TNBS-induced inflammatory bowel disease through anti-inflammation and suppressing isolated jejunum contraction. Int. J. Mol. Sci. 19 (10), E3057. 10.3390/ijms19103057 30301267 PMC6213068

[B23] Heinrich M. Appendino G. Efferth T. Fürst R. Izzo A. A. Kayser O. (2020). Best practice in research - overcoming common challenges in phytopharmacological research. J. Ethnopharmacol. 246, 112230. 10.1016/j.jep.2019.112230 31526860

[B24] Higgins J. P. Altman D. G. Gøtzsche P. C. Jüni P. Moher D. Oxman A. D. (2011). The Cochrane Collaboration's tool for assessing risk of bias in randomised trials. Bmj 343, d5928. 10.1136/bmj.d5928 22008217 PMC3196245

[B25] Hu J. Li P. Zhang T. (2018). Rhubarb combined with trypsin inhibitor for severe acute pancreatitis: A systematic review and meta-analysis. Phytother. Res. 32 (8), 1450–1458. 10.1002/ptr.6096 29672966

[B26] Jia Y. Wang Z. Z. Li X. M. Miao M. S. (2021). Data mining of drug use rules based on traditional Chinese medicine treatment of ulcerative colitis. Zhongguo Zhong Yao Za Zhi 46 (10), 2594–2600. 10.19540/j.cnki.cjcmm.20200911.501 34047108

[B27] Kaplan G. G. (2015). The global burden of IBD: from 2015 to 2025. Nat. Rev. Gastroenterol. Hepatol. 12 (12), 720–727. 10.1038/nrgastro.2015.150 26323879

[B28] Kim D. H. (2018). Gut microbiota-mediated pharmacokinetics of ginseng saponins. J. Ginseng Res. 42 (3), 255–263. 10.1016/j.jgr.2017.04.011 29983606 PMC6026358

[B29] Kim E. S. Kim W. H. (2010). Inflammatory bowel disease in korea: epidemiological, genomic, clinical, and therapeutic characteristics. Gut Liver 4 (1), 1–14. 10.5009/gnl.2010.4.1.1 20479907 PMC2871616

[B30] Ko C. W. Singh S. Feuerstein J. D. Falck-Ytter C. Falck-Ytter Y. Cross R. K. (2019). AGA clinical practice guidelines on the management of mild-to-moderate ulcerative colitis. Gastroenterology 156 (3), 748–764. 10.1053/j.gastro.2018.12.009 30576644 PMC6858922

[B31] Kobayashi T. Siegmund B. Le Berre C. Wei S. C. Ferrante M. Shen B. (2020). Ulcerative colitis. Nat. Rev. Dis. Prim. 6 (1), 74. 10.1038/s41572-020-0205-x 32913180

[B32] Li R. (2019). Analysis of curative effect and prognosis of modified Shaoyao decoction retention enema in the treatment of chronic ulcerative colitis. Sichuan J. TCM 37 (9), 95–98.

[B33] Li Z. M. (2012). Shaoyao decoction with olsalazine sodium in the treatment of damp and activity of ulcerative colitis control study. J. Pract. Traditional Chin. Intern. Med. 26 (5), 74–75. 10.3969/j.issn.1671-7813.2012.05.38

[B34] Li Z. W. Niu D. L. Zhao S. W. (2021). Clinical observation of 43 cases of ulcerative colitis treated with combined Chinese and Western medicine. Chin. J. Ethnomedicine Ethnopharmacy 30 (15), 84–86.

[B35] Lipinski S. Bremer L. Lammers T. Thieme F. Schreiber S. Rosenstiel P. (2011). Coagulation and inflammation. Molecular insights and diagnostic implications. Hamostaseologie 31 (2), 94104–95102. 10.5482/ha-1134 21152678

[B36] Liu Y. H. (2019). Effect of Jiawei Shaoyao Decoction retention enema on damp-heat ulcerative colitis and its effect on inflammatory factors. Guangxi J. TCM 42 (2). 10.3969/j.issn.1003-0719.2019.02.010

[B37] Liu Y. Yin H. Chen K. (2013). Platelet proteomics and its advanced application for research of blood stasis syndrome and activated blood circulation herbs of Chinese medicine. Sci. China. Life Sci. 56 (11), 1000–1006. 10.1007/s11427-013-4551-8 24114444

[B38] Miao B. Li F. W. Zhang S. W. Wang H. Qi W. J. Wang C. (2018). Efficacy and safety of tongfu powder in acute pancreatitis patients with gastrointestinal dysfunction: a clinical trial. Drug Des. devel. Ther. 12, 3665–3673. 10.2147/dddt.S163645 PMC6217005 30464398

[B39] Moher D. Liberati A. Tetzlaff J. Altman D. G. (2009). Preferred reporting items for systematic reviews and meta-analyses: the PRISMA statement. Bmj 339, b2535. 10.1136/bmj.b2535 19622551 PMC2714657

[B40] Molodecky N. A. Soon I. S. Rabi D. M. Ghali W. A. Ferris M. Chernoff G. (2012). Increasing incidence and prevalence of the inflammatory bowel diseases with time, based on systematic review. Gastroenterology 142 (1), 46–54. 10.1053/j.gastro.2011.10.001 22001864

[B41] Naini M. A. Zargari-Samadnejad A. Mehrvarz S. Tanideh R. Ghorbani M. Dehghanian A. (2021). Anti-inflammatory, antioxidant, and healing-promoting effects of Aloe vera extract in the experimental colitis in rats. Evid. Based. Complement. Altern. Med. 2021, 9945244. 10.1155/2021/9945244 PMC8668319 34912469

[B42] Neurath M. F. (2014). Cytokines in inflammatory bowel disease. Nat. Rev. Immunol. 14 (5), 329–342. 10.1038/nri3661 24751956

[B43] Nong Z. B. (2009). Clinical observation of 30 cases of ulcerative colitis treated with oral sulfasalazine plus traditional Chinese medicine retention enema. Intern. Med. 4 (6), 879–880. 10.16121/j.cnki.cn45-1347/r.2009.06.085

[B44] Pedersen J. LaCasse E. C. Seidelin J. B. Coskun M. Nielsen O. H. (2014). Inhibitors of apoptosis (IAPs) regulate intestinal immunity and inflammatory bowel disease (IBD) inflammation. Trends Mol. Med. 20 (11), 652–665. 10.1016/j.molmed.2014.09.006 25282548

[B45] Pepys M. B. Hirschfield G. M. (2003). C-reactive protein: a critical update. J. Clin. Invest. 111 (12), 1805–1812. 10.1172/jci18921 12813013 PMC161431

[B46] Qin Y. Wang J. B. Kong W. J. Zhao Y. L. Yang H. Y. Dai C. M. (2011). The diarrhoeogenic and antidiarrhoeal bidirectional effects of rhubarb and its potential mechanism. J. Ethnopharmacol. 133 (3), 1096–1102. 10.1016/j.jep.2010.11.041 21112382

[B47] Reinisch W. Reinink A. R. Higgins P. D. (2015). Factors associated with poor outcomes in adults with newly diagnosed ulcerative colitis. Clin. Gastroenterol. Hepatol. 13 (4), 635–642. 10.1016/j.cgh.2014.03.037 24887059

[B48] Sehgal P. Colombel J. F. Aboubakr A. Narula N. (2018). Systematic review: safety of mesalazine in ulcerative colitis. Aliment. Pharmacol. Ther. 47 (12), 1597–1609. 10.1111/apt.14688 29722441

[B49] Seo E. J. Ngoc T. M. Lee S. M. Kim Y. S. Jung Y. S. (2012). Chrysophanol-8-O-glucoside, an anthraquinone derivative in rhubarb, has antiplatelet and anticoagulant activities. J. Pharmacol. Sci. 118 (2), 245–254. 10.1254/jphs.11123fp 22302018

[B50] Sheng R. D. Shi L. P. Zhang J. L. Zheng F. Yang D. Q. Ran M. F. (2017). Modified peony decoction combined with mesalazine on ulcerative colitis (internal dampness-heat syndrome type). J. Emerg. Traditional Chin. Med. 26 (9), 1619–1622. 10.3969/j.issn.1004-745X.2017.09.035

[B51] Shi A. P. Sun A. X. (2017). Observation on the curative effect of Kangfuxin liquid combined with self-made Shaoyao decoction assisted by olsalazine in the treatment of ulcerative colitis (damp-heat syndrome). J. Emerg. Traditional Chin. Med. 26 (12), 2235–2236. 10.3969/j.issn.1004-745X.2017.12.052

[B52] Smyth S. S. McEver R. P. Weyrich A. S. Morrell C. N. Hoffman M. R. Arepally G. M. (2009). Platelet functions beyond hemostasis. J. Thromb. Haemost. 7 (11), 1759–1766. 10.1111/j.1538-7836.2009.03586.x 19691483

[B53] Sood A. Midha V. Sood N. Bhatia A. S. Avasthi G. (2003). Incidence and prevalence of ulcerative colitis in Punjab, North India. Gut 52 (11), 1587–1590. 10.1136/gut.52.11.1587 14570727 PMC1773859

[B54] Sun J. X. Zhang X. A. (2020). Discussion on the clinical study on yang-warming and qi-benefiting and Detoxifi⁃cating prescription for ulcerative colitis based on the theory of "toxic cold. J. New Chin. Med. 52 (2), 72–73. 10.13457/j.cnki.jncm.2020.02.020

[B55] Tan G. Z. Sun J. Qun Y. Z. Zhou F. Pei C. Feng T. (2020). Clinical observation of Shaoyao decoction and infliximab on moderate or severe ulcerative colitis. Shanxi J. TCM 36 (7), 23–26. 10.3969/j.issn.1000-7156.2020.07.009

[B56] Teng J. L. Zhang Y. X. Wu Q. G. Chen Z. Huang X. W. (2019). Chinese Materia medica. China: People's Medical Publishing House, 106–107.

[B57] Tian G. D. (2017). Clinical effect of modified Shaoyao decoction in the treatment of ulcerative colitis. Neimenggu J. TCM 36 (17), 3–4. 10.3969/j.issn.1006-0979.2017.17.003

[B58] Ungaro R. Mehandru S. Allen P. B. Peyrin-Biroulet L. Colombel J. F. (2017). Ulcerative colitis. Lancet 389 (10080), 1756–1770. 10.1016/s0140-6736(16)32126-2 27914657 PMC6487890

[B59] Waldner M. J. Neurath M. F. (2014). Master regulator of intestinal disease: IL-6 in chronic inflammation and cancer development. Semin. Immunol. 26 (1), 75–79. 10.1016/j.smim.2013.12.003 24447345

[B60] Wang C. S. Li W. B. Wang H. Y. Ma Y. M. Zhao X. H. Yang H. (2018). VSL#3 can prevent ulcerative colitis-associated carcinogenesis in mice. World J. Gastroenterol. 24 (37), 4254–4262. 10.3748/wjg.v24.i37.4254 30310258 PMC6175759

[B61] Wang F. T. (2016). Influence of ZhiKang capsules on serum TNF-α, IL-6 and CRP of the patients with ulcerative colitis. West. J. Traditional Chin. Med. 29 (2), 100–102. 10.3969/j.issn.1004-6852.2016.02.031

[B62] Wang H. S. Shi L. P. Zhao F. L. Wu H. X. Yang D. Q. Ran M. F. (2017). Clinical effect of jiawei shaoyao decoction in treatment of ulcerative colitis with internal retention of damp-heat: an analysis of 32 cases. Hunan J. TCM 33 (11), 11–14. 10.16808/j.cnki.issn1003-7705.2017.11.004

[B63] Wang J. X. (2018). Observation of curative effect of modified Lishihexue decoction combined with Bupi Yichang Pill in the treatment of 48 cases of ulcerative colitis. Asia-Paeitic Tradit. Med. 14 (3). 10.11954/ytctyy.201803068

[B64] Wang P. L. (2020). Treatment of ulcerative colitis with Shaoyao Decoction and Gegen Qinlian Decoction (Intestinal damp-heat syndrome) clinical curative effect observation. Sichuan J. TCM 38 (1), 101–103.

[B65] Wang S. Wang J. Ma R. Yang S. Fan T. Cao J. (2020). IL-10 enhances T cell survival and is associated with faster relapse in patients with inactive ulcerative colitis. Mol. Immunol. 121, 92–98. 10.1016/j.molimm.2020.03.001 32193038

[B66] Wen B. Sun J. P. (2017). Efficacy observation of Zhikang capsule combined with mesalazine in the treatment of mild to moderate ulcerative colitis. Hainan Med. J. 28 (23), 3820–3822. 10.3969/j.issn.1003-6350.2017.23.012

[B67] Windsor A. Michetti P. Bemelman W. Ghosh S. (2013). The positioning of colectomy in the treatment of ulcerative colitis in the era of biologic therapy. Inflamm. Bowel Dis. 19 (12), 2695–2703. 10.1097/MIB.0b013e318292fae6 23846487

[B68] Wu J. Wei Z. Cheng P. Qian C. Xu F. Yang Y. (2020). Rhein modulates host purine metabolism in intestine through gut microbiota and ameliorates experimental colitis. Theranostics 10 (23), 10665–10679. 10.7150/thno.43528 32929373 PMC7482825

[B69] Xiang H. Zuo J. Guo F. Dong D. (2020). What we already know about rhubarb: a comprehensive review. Chin. Med. 15, 88. 10.1186/s13020-020-00370-6 32863857 PMC7448319

[B70] Xie Q. Li H. Ma R. Ren M. Li Y. Li J. (2022). Effect of Coptis chinensis franch and Magnolia officinalis on intestinal flora and intestinal barrier in a TNBS-induced ulcerative colitis rats model. Phytomedicine. 97, 153927. 10.1016/j.phymed.2022.153927 35030387

[B71] Xue H. C. Xu Y. (2021). Clinical effect of jiawei shaoyao decoction on ulcerative colitis. Inn. Mong. J. TCM 40 (1), 35–36. 10.16040/j.cnki.cn15-1101.2021.01.021

[B72] Yang M. M. Luo W. P. Li K. Y. Wang Q. Z. (2017). Clinical effect of retention enema with qinbai granules in treatment of ulcerative colitis with damp-heat accumulation: an analysis of 37 cases. Hunan J. TCM 33 (12), 10–12. 10.16808/j.cnki.issn1003-7705.2017.12.004

[B73] Yang X. Q. Wang Y. (2021). Clinical observation and mechanism study of pingkuiyichang recipe in treating ulcerative colitis. Hubei J. TCM 43 (2), 3–5.

[B74] Yin P. Li W. Yang H. M. Dong M. Song J. Y. (2021). Clinical study on retention enema with Jiawei Shaoyao Decoction and Kangfuxin Liquid combined with oral administration of mesalazine enteric-coated tablets in the treatment of ulcerative colitis of large intestine damp-heat type. Hebei J. TCM 43 (2), 278–279. 10.3969/j.issn.1002-2619.2021.02.023

[B75] Yoshida H. Granger D. N. (2009). Inflammatory bowel disease: a paradigm for the link between coagulation and inflammation. Inflamm. Bowel Dis. 15 (8), 1245–1255. 10.1002/ibd.20896 19253306 PMC2713811

[B76] You C. M. Shen Y. H. Shen T. C. (2015). Clinical observation on 42 cases of ulcerative colitis treated with modified Gancao Xiexin decoction. Neimenggu J. TCM 35 (01), 18–19. 10.16040/j.cnki.cn15-1101.2016.01.018

[B77] Yuan X. H. (2020). Effects of Zhikang Capsules combined with mesalazine on inflammatory indexes in patients with mild to moderate ulcerative colitis. Med. Forum 24 (29), 4240–4241. 10.19435/j.1672-1721.2020.29.060

[B78] Zhang C. Li J. Xiao M. Wang D. Qu Y. Zou L. (2022a). Oral colon-targeted mucoadhesive micelles with enzyme-responsive controlled release of curcumin for ulcerative colitis therapy. Chin. Chem. Lett. 33 (11), 4924–4929. 10.1016/j.cclet.2022.03.110

[B79] Zhang C. Wang X. Xiao M. Ma J. Qu Y. Zou L. (2022b). Nano-in-micro alginate/chitosan hydrogel via electrospray technology for orally curcumin delivery to effectively alleviate ulcerative colitis. Mater. Des. 221, 110894. 10.1016/j.matdes.2022.110894

[B86] Zhang G. R. (2020). Effect and mechanism of shaoyao decoction in the treatment of patients with ulcerative colitis. China J. Pharmaceut. Econ. 15 (11), 100–103. 10.12010/j.issn.1673-5846.2020.11.025

[B80] Zhang H. Y. (2016). Observation on the therapeutic effect of banxiaxiexin decoction in the treatment of 40 cases of ulcerative colitis. Asia-Paeitic Tradit. Med. 12 (2), 109–110. 10.11954/ytctyy.201602052

[B81] Zhang L. Wu Q. Chen P. Xu Y. Zheng Y. Ma S. (2021). Effect of tongfu traditional Chinese medicine preparation on patients with septic gastrointestinal dysfunction: a systematic review and meta-analysis. Ann. Palliat. Med. 10 (12), 12072–12085. 10.21037/apm-21-2461 35016408

[B82] Zhang Y. L. Cai L. T. Qi J. Y. Lin Y. Z. Dai Y. C. Jiao N. (2019). Gut microbiota contributes to the distinction between two traditional Chinese medicine syndromes of ulcerative colitis. World J. Gastroenterol. 25 (25), 3242–3255. 10.3748/wjg.v25.i25.3242 31333315 PMC6626730

[B83] Zhao J. H. (2020). Effect of Changwei'an pill combined with mesalazine on serum inflammatory index levels in patients with ulcerative colitis. J. Chende Med. Coll. 37 (6), 487–490. 10.15921/j.cnki.cyxb.2020.06.011

[B84] Zhao Y. Q. Liu X. H. Ito T. Qian J. M. (2004). Protective effects of rhubarb on experimental severe acute pancreatitis. World J. Gastroenterol. 10 (7), 1005–1009. 10.3748/wjg.v10.i7.1005 15052683 PMC4717089

[B85] Zhou Y. Wang L. Huang X. Li H. Xiong Y. (2016). Add-on effect of crude rhubarb to somatostatin for acute pancreatitis: A meta-analysis of randomized controlled trials. J. Ethnopharmacol. 194, 495–505. 10.1016/j.jep.2016.09.053 27693773

