# Supplementary material for: The application of Tong-fu therapeutic method on ulcerative colitis: A systematic review and meta-analysis for efficacy and safety of rhubarb-based therapy
Source: Front Pharmacol. 2022 Oct 21;13:1036593. doi: 10.3389/fphar.2022.1036593 (PMC9634183; doi:10.3389/fphar.2022.1036593)
Supplement: Supplementary file 1 [file DataSheet1.docx]

Supplementary Material

# Supplementary Data

**PubMed Search formation**

(("Colitis, Ulcerative"[Mesh]) OR ((((Idiopathic Proctocolitis[Title/Abstract]) OR (Ulcerative Colitis[Title/Abstract])) OR (Colitis Gravis[Title/Abstract])) OR (Inflammatory Bowel Disease, Ulcerative Colitis Type[Title/Abstract]))) AND (("Rheum"[Mesh]) OR ((((((((Rheum tanguticum) OR (Rheum officinale)) OR (Da Huang)) OR (Huang, Da)) OR (Chinese Rhubarb)) OR (Rhubarb, Chinese)) OR (Rhubarb)) OR (Rheum rhaponticum)))

**EMBASE Search formation**

#17. #15 AND #16

#16. #6 OR #7 OR #8 OR #9 OR #10 OR #11 OR #12 OR #13

OR #14

#15. #1 OR #2 OR #3 OR #4 OR #5

#14. rheum AND rhaponticum

#13. rhubarb

#12. rhubarb, AND chinese

#11. chinese AND rhubarb

#10. huang, AND da

#9. da AND huang

#8. rheum AND officinale

#7. rheum AND tanguticum

#6. 'rheum'/exp

#5. inflammatory AND bowel AND disease, AND

ulcerative AND colitis AND type:ab,ti

#4. colitis AND gravis:ab,ti

#3. ulcerative AND colitis:ab,ti

#2. idiopathic AND proctocolitis:ab,ti

#1. 'ulcerative colitis'/exp AND +

**Cochrane search formation**

#1 MeSH descriptor: [Colitis, Ulcerative] explode all trees

#2 (Idiopathic Proctocolitis):ti,ab,kw OR (Ulcerative Colitis):ti,ab,kw OR (Colitis Gravis):ti,ab,kw OR (Inflammatory Bowel Disease, Ulcerative Colitis Type):ti,ab,kw (Word variations have been searched)

#3 MeSH descriptor: [Rheum] explode all trees

#4 (Rheum tanguticum) OR (Rheum officinale) OR (Da Huang) OR (Huang, Da) OR (Chinese Rhubarb) (Word variations have been searched)

#5 (Rhubarb, Chinese) OR (Rhubarb) OR (Rheum rhaponticum) (Word variations have been searched)

#6 #1 OR #2

#7 #3 OR #4 OR #5

#8 #6 AND #7

**CNKI search formation**

(SU=溃疡性结肠炎 OR TKA=溃疡性结肠炎) AND (SU=大黄 OR TKA=大黄 OR FT=大黄)

**Wanfang database search formation**

（主题=溃疡性结肠炎）AND 大黄

**CBM search formation**

(“溃疡性结肠炎”[常用字段] AND “大黄” [全部字段])

**VIP search formation**

(M=溃疡性结肠炎 OR R=溃疡性结肠炎) AND (M=大黄 OR R=大黄 OR U=大黄)

# Supplementary Table

## Supplementary Table S1

# The characteristics of included study in this meta-analysis.

| **Studies** | **Sample size** | **Interventions** | | **Drug delivery** | | **Course** | **Outcome** |
| --- | --- | --- | --- | --- | --- | --- | --- |
|  | (T/C *n*) | T | C |  |  |  |  |
| Chen B.Q.2020 | 86 (43/43) | SASP+ rhubarb-based  medicinal formula | SASP | | Oral | 13W | aghlm |
| Deng P.2015 | 72 (36/36) | SASP+ rhubarb-based  medicinal formula | SASP | | Oral + Enema | 4W | a |
| Deng S.H.2020 | 90 (45/45) | 5-ASA + rhubarb-based  medicinal formula | 5-ASA | | Oral | 2W | a |
| Ding S.L.2016 | 90 (45/45) | SASP+ rhubarb-based  medicinal formula | SASP | | Enema | 8W | a |
| Fei X.Y.2017 | 150 (75/75) | 5-ASA + rhubarb-based  medicinal formula | 5-ASA | | Oral | 9W | acd |
| Guo G.J.2019 | 50 (25/25) | 5-ASA + rhubarb-based  medicinal formula | 5-ASA | | Oral | 8W | ae |
| Li R.2019 | 92 (46/46) | 5-ASA + rhubarb-based  medicinal formula | 5-ASA | | Enema | 4W | aeij |
| Liu Y.H.2019 | 81 (41/40) | 5-ASA + rhubarb-based  medicinal formula | 5-ASA | | Enema | 4W | aghi |
| Li Z.M.2012 | 40 (20/20) | OS + rhubarb-based  medicinal formula | OS | | Oral | 6W | a |
| Li Z.W.2021 | 86 (43/43) | 5-ASA + rhubarb-based  medicinal formula | 5-ASA | | Oral | 8W | bcdn |
| Nong Z.B.2009 | 60 (30/30) | SASP+ rhubarb-based  medicinal formula | SASP | | Enema | 3W | a |
| Sheng R.D.2017 | 96 (48/48) | 5-ASA + rhubarb-based  medicinal formula | 5-ASA | | Oral | 4W | am |
| Shi A.P.2017 | 100 (50/50) | OS + rhubarb-based  medicinal formula | OS | | Enema | 2W | acgi |
| Sun J.X.2020 | 120 (60/60) | BB + GM + 5-ASA+ rhubarb-based  medicinal formula | BB + GM + 5-ASA | | Oral | 2W | agl |
| Tan G.Z.2020 | 68 (34/34) | IFX + rhubarb-based  medicinal formula | IFX | | Oral | 12W | abdfghj |
| Tian G.D.2017 | 73 (38/35) | rhubarb-based  medicinal formula | 5-ASA | | Oral | 8W | aen |
| Wang F.T.2016 | 58 (29/29) | PS + SASP + rhubarb based medicinal formula | PS + SASP | | Oral | 2W | afghk |
| Wang H.S.2017 | 64 (32/32) | 5-ASA + rhubarb-based  medicinal formula | 5-ASA | | Oral | 4W | am |
| Wang J.X.2018 | 95 (48/47) | 5-ASA + rhubarb-based  medicinal formula | 5-ASA | | Oral | 8W | adgim |
| Wang P.L.2020 | 96 (48/48) | 5-ASA + rhubarb-based  medicinal formula | 5-ASA | | Oral | 4W | aghi |
| Wen B.2017 | 98 (49/49) | 5-ASA + rhubarb-based  medicinal formula | 5-ASA | | Oral | 4W | aghjl |
| Xue H.C.2021 | 64 (32/32) | SASP+ rhubarb-based  medicinal formula | SASP | | Oral | 8W | ahi |
| Yang M.M.2017 | 73 (37/36) | 5-ASA + rhubarb-based  medicinal formula | 5-ASA | | Enema | 12W | a |
| Yang X.Q.2021 | 90 (45/45) | rhubarb-based  medicinal formula | 5-ASA | | Oral + Enema | 4W | aghi |
| Yin P.2021 | 100 (50/50) | 5-ASA + rhubarb-based  medicinal formula | HCSS + 5-ASA | | Enema | 8W | abfgiklm |
| You C.M.2016 | 84 (42/42) | rhubarb-based  medicinal formula | 5-ASA | | Oral | 8W | a |
| Yuan X.H.2020 | 63 (31/32) | 5-ASA + rhubarb-based  medicinal formula | 5-ASA | | Oral | 2W | aghk |
| Zhang G.R.2020 | 98 (49/49) | 5-ASA + rhubarb-based  medicinal formula | 5-ASA | | Oral | 8W | agn |
| Zhang H.Y.2016 | 80 (40/40) | rhubarb-based  medicinal formula | SASP | | Oral | 8W | am |
| Zhao J.H.2020 | 90 (45/45) | 5-ASA + rhubarb-based  medicinal formula | 5-ASA | | Oral | 12W | aghkl |

Abbreviation in Table S1: (T) Treatment group; (C) Control group; (5-ASA) 5-aminosalicylic acid; (SASP) Sulfasalazine; (OS) Olsalazine sodium; (BB) Bifidobacterium triple viable capsules; (GM) Glutamine; (IFX) Infliximab; (PS) Prednisolone; (HCSS) Hydrocortisone sodium succinate; (NR) not reported; (a) Clinical effective rate; (b) MAYO score; (c) Geboes score; (d) Baron score; (e) Recurrence rate; (f) PLT; (g) TNF-α; (h) IL-6; (i) IL-8; (j) IL-10; (k) CRP; (l) IL-1β; (m) Adverse events; (n) TCM symptoms integral.

# Supplementary Figure

## Supplementary Figure S1


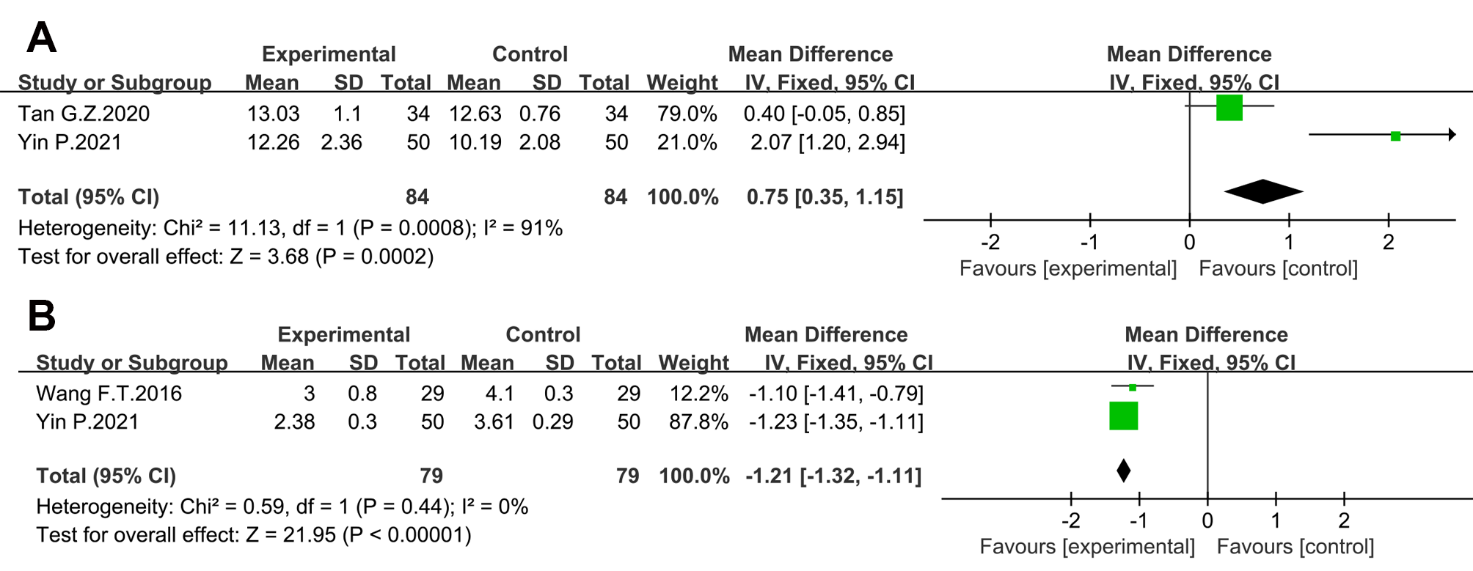


**Supplementary Figure S1.** Forest plot showing the result of (A) PT, (B) FIB.

## Supplementary Figure S2


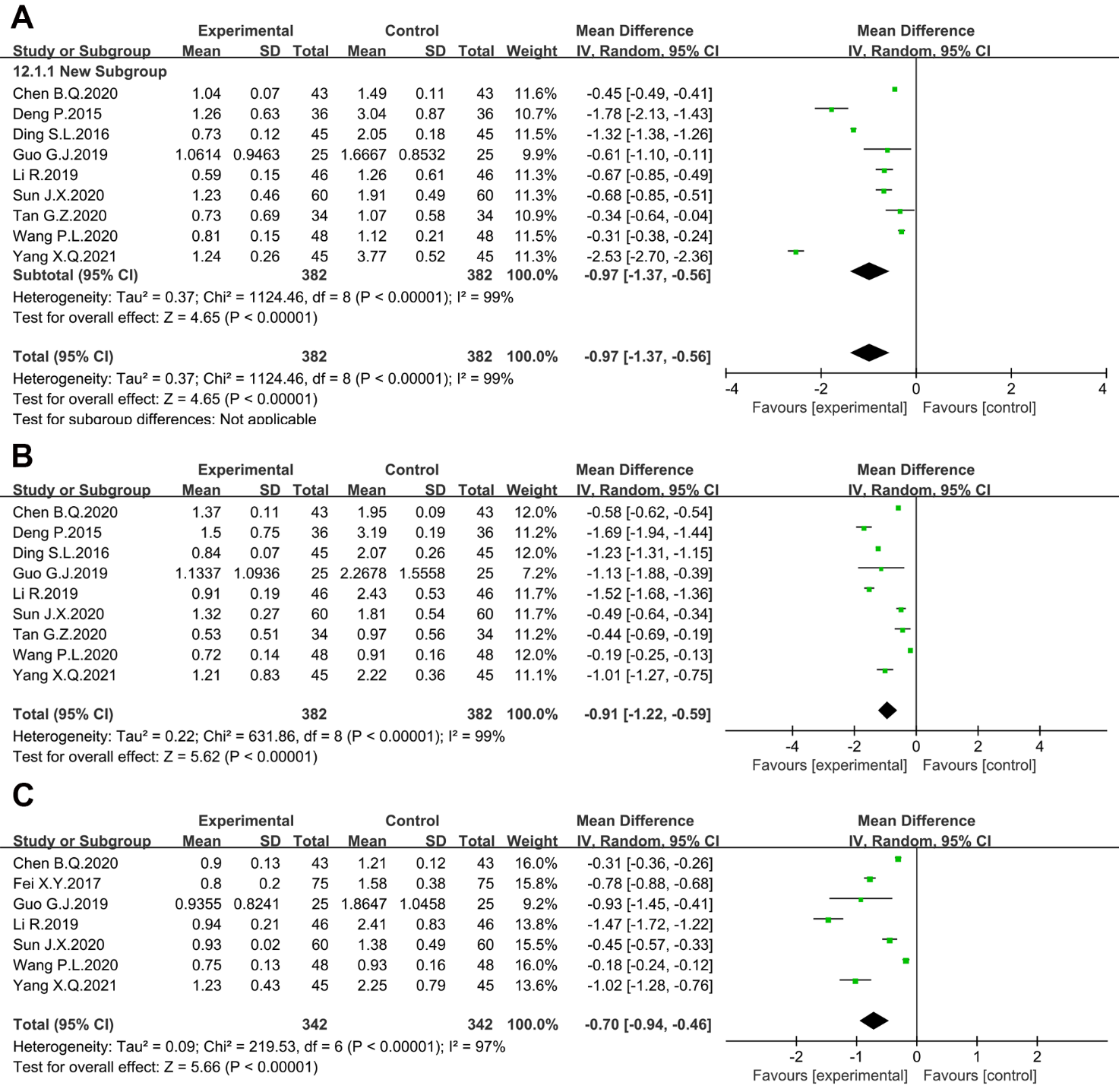
**Supplementary Figure S2.** Forest plot showing the result of the individual symptom scores of (A) abdominal pain, (B) diarrhea., (C) urgency.

## Supplementary Figure S3


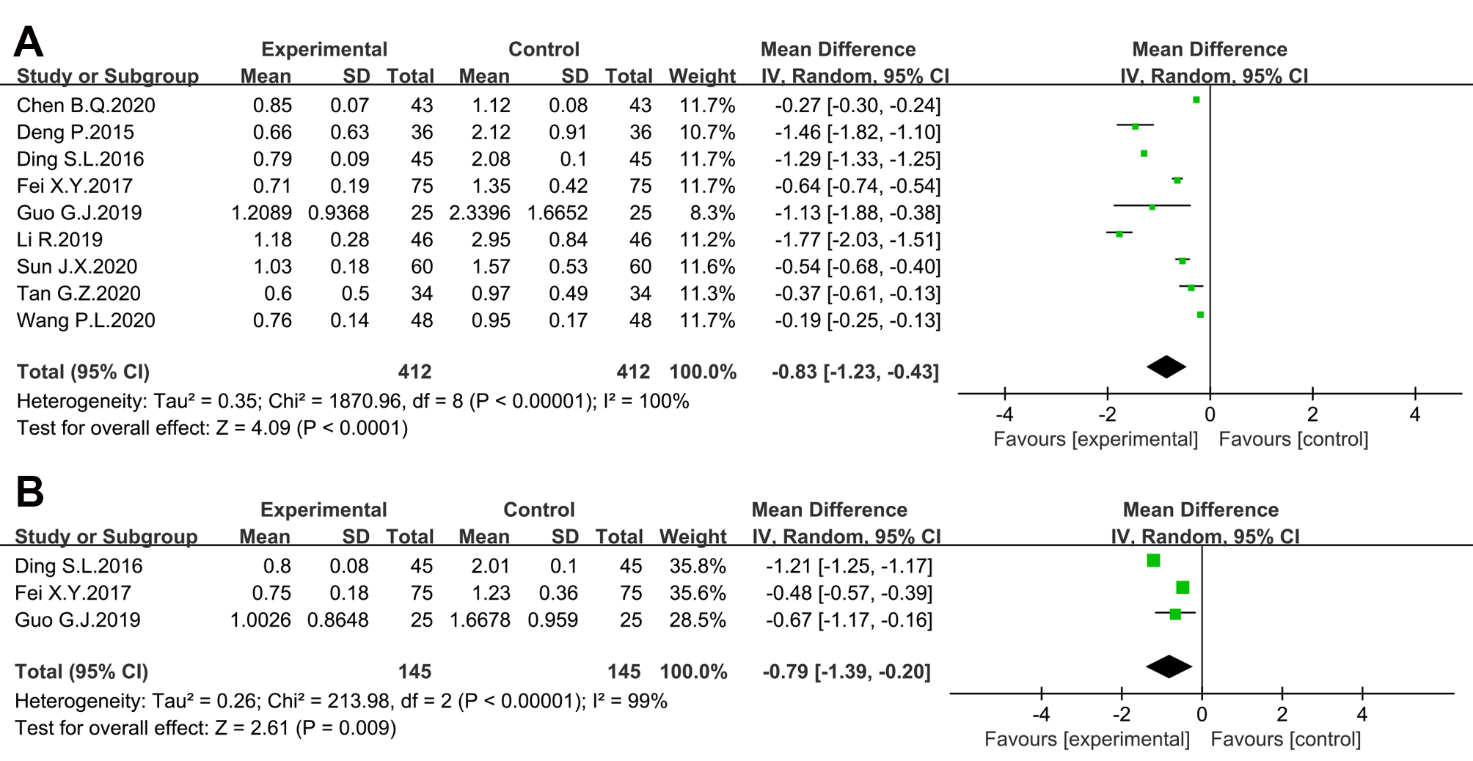


**Supplementary Figure S3.** Forest plot showing the result of the individual symptom scores of (A) pus and blood stool, (B) burning pain in the anus.

## Supplementary Figure S4


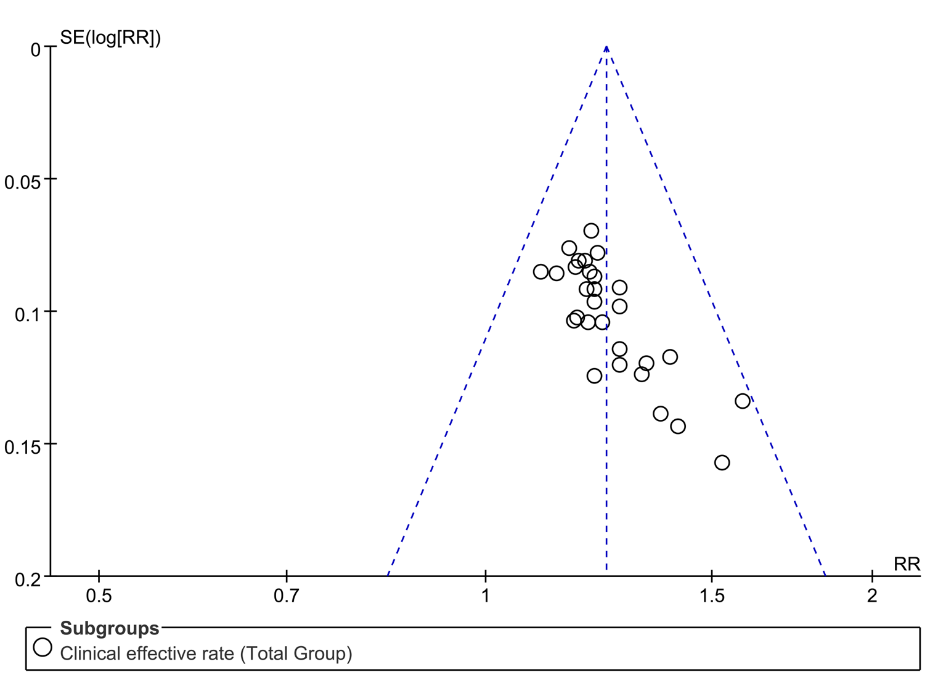


**Supplementary Figure S4.** Funnel plot to assess publication bias for primary outcome in this Meta-analysis..

##
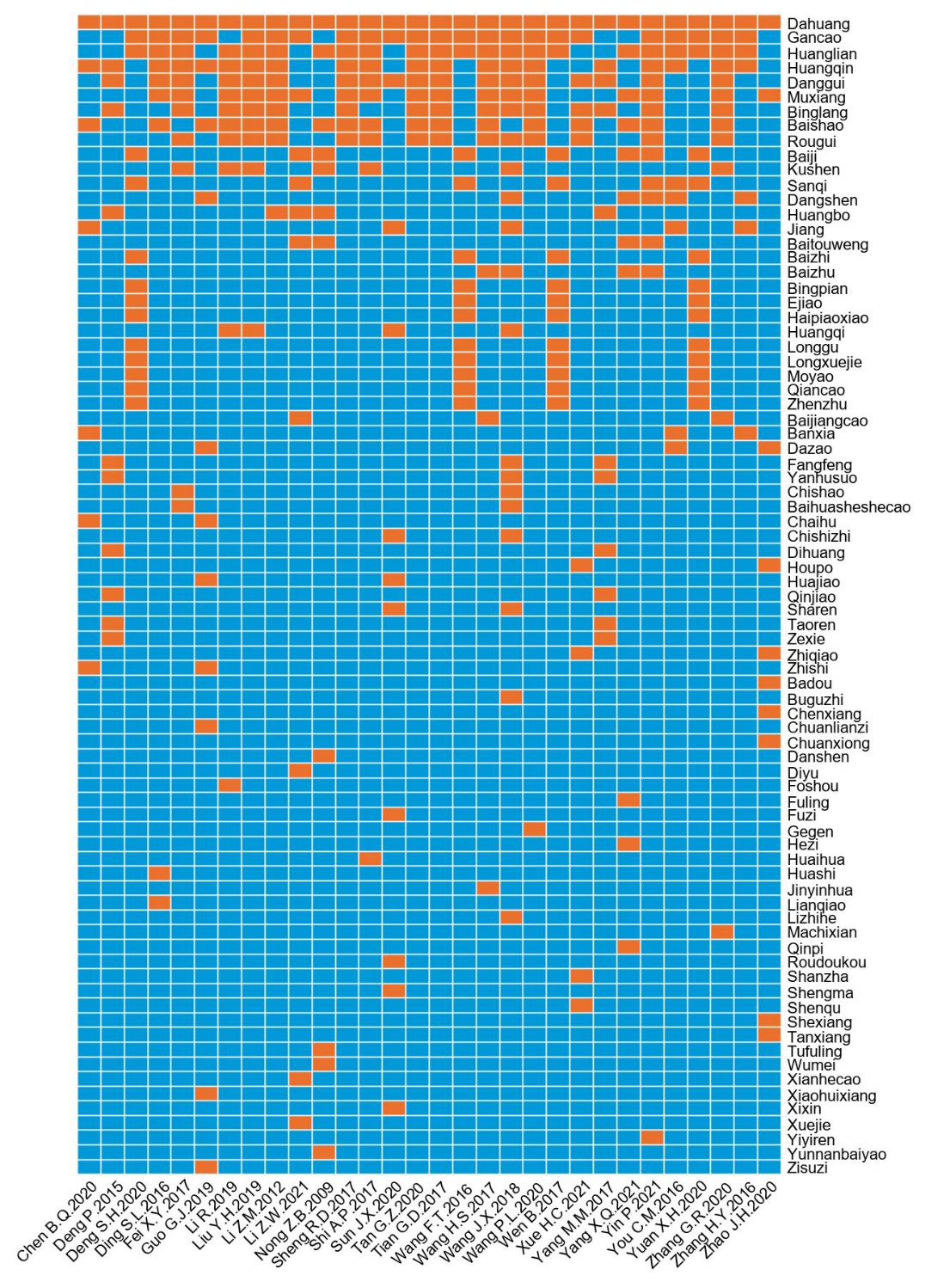
Supplementary Figure S5

**Supplementary Figure S5.** Data regarding Rhubarb-based TCM formulations.

Abbreviation in Figure S5: Dahuang (the dried root and rhizome of *Rheum palmatum* L.), Gancao (the dried root and rhizome of *Glycyrrhiza glabra* L.), Huanglian (the dried rhizome of *Coptis chinensis* Franch.), Huangqin (the dried root of *Scutellaria baicalensis* Georgi), Danggui (the dried root of *Angelica sinensis* (Oliv.) Diels), Muxiang (the dried root of *Dolomiaea costus (Falc.) Kasana & A.K.Pandey*), Binglang (the dry ripening of *Areca catechu* L.), Baishao (the dried root of *Paeonia lactiflora* Pall.), Rougui (the dried bark of *Neolitsea cassia (L.) Kosterm.*), Baiji (the dried tubers of *Bletilla striata* (Thunb.) Reichb.f.), Kushen (the dried root of *Sophora flavescens* Ait.), Sanqi (the dried root and rhizome of *Panax notoginseng* (Burk.) F. H. Chen), Dangshen (the dried root of *Codonopsis pilosula* (Franch.)Nannf.), Huangbo (the dried bark of *Phellodendron chinense* Schneid.), Jiang (the rhizome of *Zingiber officinale* Rosc.), Baitouweng(the dried root of *Pulsatilla chinensis* (Bge.) Regel), Baizhi (the dried root of *Angelica dahurica* (Fisch，ex Hoffm.) Benth.et Hook.f.), Baizhu (the dried rhizome of *Atractylodes macrocephala* Koidz.), Bingpian (Borneolum Syntheticum), Ejiao (the solid glue made from the dried skin of *Equus asinus* L.), Haipiaoxiao (the dry inner shell of *SepielLa maindroni* deRochebrune), Huangqi (the dried root of *Astragalus membranaceus* (Fisch.) Bge.var.mongholicus (Bge.) Hsiao), Longgu (Fossilia Ossis Mastodi), Longxuejie (the dry resin *Resina* Draconis.), Moyao (the dry resin of *Commiphora myrrha* Engl.), Qiancao (the dried root and rhizome of *Rubia cordifolia* L.), Zhenzhu (the stimulated pearls formed by bivalves of *Pteria martensii* (Dunker) ), Baijiangcao (the whole grass of *Patrinia villosa* Juss.), Banxia (the dried tuber of P*inellia ternata* (Thunb.) Breit.), Dazao (the dry ripe fruit of *Ziziphus jujuba* Mill.), Fangfeng (the dried root of *Saposhnikovia divaricata* (Turcz.) Schischk.), Yanhusuo (the dried tuber of Corydalis yanhusuo W.T.Wang), Chishao (the dried root of *Paeonia lactiflora* Pall.), Baihuasheshecao (the whole grass of *Oldenlandia diffusa* (Willd.) Roxb.), Chaihu (the dried root of *Bupleurum chinense* DC.), Chishizhi (Halloysitum Rubrum), Dihuang (the fresh or dried root of *Rehmannia glutinosa* Libosch.), Houpo (the dried bark, root bark and branch bark of *Magnolia officinalis* Rehd.et Wils.), Huajiao (the dry ripe fruit of of *Zanthoxylum schinifolium* Sieb. et Zucc.), Qinjiao (the dried root of *Gentiana macrophylla* Pall.), Sharen (the dry ripe fruit of *Amomum villosum* Lour.), Taoren (the dry ripe seed of *Prunus persica* (L.) Batsch), Zexie (the dried tuber of *Alisma orientale* (Sam.) Juzep.), Zhiqiao (the dry unripe fruit of *Citrus aurantium* L.), Zhishi (the dried young fruit of *Citrus aurantium* L.), Badou (the dry ripe fruit of *Croton tiglium* L.), Buguzhi (the dry ripe fruit of *Psoralea corylifolia* L.), Chenxiang (the resinous wood of *Aquilaria sinensis* ( Lour.) Gilg), Chuanlianzi (the dry ripe fruit of *Melia toosendan* Sieb.et Zucc.), Chuanxiong (the dried rhizome of *Ligusticum chuanxiong* Hort.), Danshen (the dried root and rhizome of *Salvia miltiorrhiza* Bge.), Diyu (the dried root of *Sanguisorba officinalis* L.), Foshou (the dried fruit of *Citrus medica L. var. sarco-dactylis* Swingle), Fuling (the dry sclerotia of *Poria cocos*（Schw.）Wolf), Fuzi (the processed product of *Aconitum carmichaelii* Debx.), Gegen (the dried root of *Pueraria lobata* (Willd.) Ohwi), Hezi (the dry ripe fruit of *Terminalia chebula* Retz.), Huaihua (the dried flower and bud of *Sophora japonica* L.), Jinyinhua (the dry flower bud of *Lonicera japonica* Thunb.), Lianqiao (the dried fruit of *Forsythia suspensa* (Thunb.) Vahl), Lizhihe (the dry ripe seed of *Litchi chinensis* Sonn.), Machixian (the dry above ground of *Portulaca oleracea* L.), Qinpi (the dry bark of *Fraxinus rhynchophylla* Hance), Roudoukou (the dried seed of *Myristica fragrans* Houtt.), Shanzha (the dry ripe fruit of *Crataegus pinnatifida* Bge. *var. Major* N. E. Br.), Shengma (the dry rhizome of *Cimicifuga heracleifolia* Kom.), Shenqu (Massa Medicata Fermentata), Shexiang (the dried secretion in mature male sachets *Moschus berezovskii* Flerov), Tanxiang (the dried heartwood of tree trunk *Santalum album* L.), Tufuling (the dry rhizome of *Smilax glabra* Roxb.), Wumei (the dry near-ripe fruit *Prunus mume* (Sieb.) Sieb.etZucc.), Xianhecao (the dry above ground of *Agrimonia pilosa* Ledeb.), Xiaohuixiang (the dry ripe fruit of *Foeniculum vulgare* Mill.), Xixin (the dried root and rhizome of *Asarum heterotropoides* Fr. Schmidt var. *mandshuricum* (Maxim.) Kitag.), Xuejie (the resin of *Daemonorops draco* Bl.), Yiyiren (the dry mature seed of *Coix lacryma-jobi* L.var.*mayuen* (Roman.) Stapf), Zisuzi (the dry ripe fruit of *Perilla frutescens* (L.) Britt.).
